# Hexakis(urea-O)iron Complex Salts as a Versatile Material Family: Overview of Their Properties and Applications

**DOI:** 10.1021/acsomega.3c09635

**Published:** 2024-03-04

**Authors:** Kende Attila Béres, Zoltán Homonnay, László Kótai

**Affiliations:** † Institute of Materials and Environmental Chemistry, HUN-REN Research Centre for Natural Sciences, Magyar Tudósok krt. 2., H-1117 Budapest, Hungary; ‡ Institute of Chemistry, ELTE Eötvös Loránd University, Pázmány Péter s. 1/A, H-1117 Budapest, Hungary

## Abstract

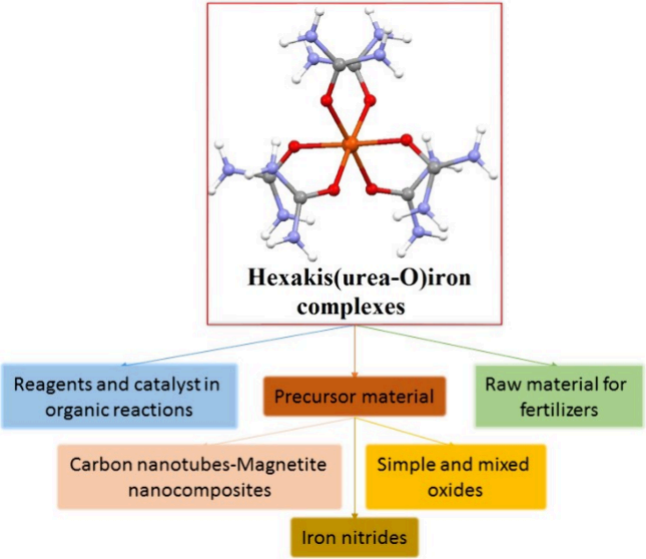

Due to their Fe- and N-containing reactive urea ligand content, the hexakis(urea-O)iron(II) and hexakis(urea-O)iron(III) complexes were found to be versatile materials in various application fields of industry and environmental protection. In our present work, we have comprehensively reviewed the synthesis, structural and spectroscopic details, and thermal properties of hexakis(urea-O)iron(II) and hexakis(urea-O)iron(III) salts with different anions (NO_3_^–^, Cl^–^, Br^–^ I^–^, I_3_^–^, ClO_4_^–^, MnO_4_^–^, SO_4_^2–^, Cr_2_O_7_^2–^, and S_2_O_8_^2–^). We compared and evaluated the structural, spectroscopic (IR, Raman, UV–vis, Mössbauer, EPR, and X-ray), and thermogravimetric data. Based on the thermal behavior of these complexes, we evaluated the solid-phase quasi-intramolecular redox reactions of anions and urea ligands in these complexes and summarized the available information on the properties of the resulting simple and mixed iron-containing oxides. Furthermore, we give a complete overview of the application of these complexes as catalysts, reagents, absorbers, or agricultural raw materials.

## 1 Introduction

The urea complexes of iron with various anions have enormous importance from scientific and industrial points of view, e.g., in the preparation of selective oxidants for organic chemistry,^1^ iron oxide-based catalysts for converting CO_2_ into hydrocarbons,^1^ nanotube–magnetite nanocomposites,^2^ various iron oxides,^3^ metallic pigments,^4^ and long-lasting trace element fertilizers.^5^ Furthermore, iron complexes with urea or urea-like linkages containing ligands ensure excellent potential to prepare bioactive complexes^6,7^ or precursor materials of different kinds of metal–organic frameworks (MOFs).^8−10^

Numerous hexacoordinated complexes containing iron(II) or iron(III) cations and urea as a ligand with different kinds of anions, including halogenides and oxometallates, were synthesized and studied first at the beginning of the 20th century.^11,12^ The O-ligation of the urea ligands in these complexes was confirmed first with IR spectroscopy.^13^ Later, a large amount of structural data (single crystal X-ray diffraction and spectroscopic) became available for most complexes. All the known and studied hexakis(urea)iron(II/III) complexes are listed in Table 1.

**Table 1:** Summary of the Known Hexakis(urea)iron(II) and Hexakis(urea-O)iron(III) Complexes

compound	label	color	melting/decomposition temperature
[Fe(urea)_6_](NO_3_)_3_	**1**	greenish-blue crystals^11^	136–139 °C^14^
[Fe(urea)_6_]Cl_3_.(3H_2_O)	**2** (**2CW**^a^)	yellow^11^	178–180 °C,^14^ 108 °C^15^
[Fe(urea)_6_]Br_3_·3H_2_O	**3**	white-greenish^11^	110 °C^15^
[Fe(urea)_6_](ClO_4_)_3_	**4**	pale bluish-green^11^	202 °C^16^
[Fe(urea)_6_](MnO_4_)_3_	**5**	purple^11^	decomposes before melting^1^
[Fe(urea)_6_]_2_(Cr_2_O_7_)_3_	**6**	yellow-orange^11^	
[Fe(urea)_6_]_2_(S_2_O_8_)_3_	**7**	bluish-green^12^	decomposes before melting^17^
[Fe(urea)_6_](NO_3_)_2_(I_3_)	**8**	red-brown^12^	
[Fe(urea)_6_]_2_(SO_4_)_3_	**9**	pale bluish^18^	
[Fe(urea)_6_]I_2_	**10**	transparent yellow^19^	145 °C^20^
[Fe(urea)_6_](I_3_)_2_	**11**	opaque shiny black^19^	
[Fe(urea)_6_](I_3_)_3_	**12**	blackish crystal^21^	
[Fe(urea)_6_]I_3_	**13**	yellow-orange^22^	

a CW = crystalline water.

The variable valence of iron, the reducing activity of urea, and the oxidation ability of the oxometallate anions triggered multidisciplinary interest, e.g., in materials science and agriculture, where these compounds are precursors in the low-temperature preparation of mixed metal oxides by anion–ligand solid-phase quasi-intramolecular redox reactions and the application of fertilizer materials with controlled N-supplementation of plant roots, respectively. Although the chemistry of similar hexaureachromium(III) complexes has been reviewed recently,^23^ the chemistry of the hexaureairon complexes has yet to be reviewed. Due to the large amount of experimental data, which sometimes are controversial, we have comprehensively reviewed the synthesis, structure, spectroscopic, and thermal properties of hexakis(urea)iron complexes, including their decomposition products and their application possibilities in various fields of chemistry and technology.

## 2 Synthesis, Composition, And Properties of the [Fe(urea)_6_]^2+/3+^ Complexes

The first examples of the hexakis(urea)iron(III) salts were synthesized by Barbieri et al.^11,12^ with Cl^–^, Br^–^, NO_3_^–^, MnO_4_^–^, Cr_2_O_7_^2–^, and S_2_O_8_^2–^ anions (Table 1). The synthesis of the complexes can be separated into two main groups.

The first synthesis route is described with eq 1, where the concentrated aqueous solution of the iron(III) salt (e.g., chloride, bromide, or nitrate) was mixed with a concentrated aqueous solution of the urea in a 1:6 molar ratio. The pH of the solutions was adjusted with the free acid of the corresponding anion (eq 1).
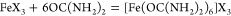
1Here, X = NO_3_^–^, Cl^–^, or Br^–^. This reaction route was used to prepare the following salts: [Fe(urea)_6_](NO_3_)_3_ (compound **1**), [Fe(urea)_6_]Cl_3_·3H_2_O (compound **2** CW), [Fe(urea)_6_]Br_3_·3H_2_O (compound **3**), and [Fe(urea)_6_](ClO_4_)_3_ (compound **4**). The same preparation method was reinvented in a patent application for compound **1** as a fertilizer material.^24^ Some compounds (X = NO_3_^–^, Cl^–^, ClO_4_^–^) were also prepared in organic solvents such as ethanol or acetone.^16,25^ However, this reaction route cannot be used for complexes, where the precursor iron(III) salts cannot be prepared easily or are very reactive, like those with anions of an oxidative nature, e.g., permanganate.^26^

The other group of hexaureairon(III) complex salts, such as permanganate or dichromate derivatives, were prepared in the metathesis reaction of a soluble and stable hexaureairon(III) salt prepared in the reaction route in eq 1 with sodium or ammonium permanganate or dichromate, respectively, according to eq 2.
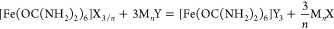
2Here, X = NO_3_– or Cl–; M = Na or NH_4_; and Y= MnO_4_ (*n* = 1), Cr_2_O_7_, or S_2_O_8_ (*n* = 2). Generally, the concentrated aqueous solution of compound **1** or **2** was mixed with the concentrated aqueous solution of the highly water-soluble (NH_4_^+^ or Na^+^) salt of the desired anions in a 1:3 (permanganate) or 1:1.5 (dichromate, persulfate) molar ratio.^1,11,12,17^

To obtain [Fe(urea)_6_](MnO_4_)_3_ (compound **5**), [Fe(urea)_6_]_2_(Cr_2_O_7_)_3_ (compound **6**), and [Fe(urea)_6_]_2_(S_2_O_8_)_3_ (compound **7**), a concentrated aqueous solution of compound **1** and NaMnO_4_, Na_2_Cr_2_O_7_, and (NH_4_)_2_S_2_O_8_ were mixed, respectively.^11,12^ Compounds **5** and **7** precipitated immediately upon mixing the solutions, while compound **6** could be isolated after concentrating the solution by evaporation.^11,12^ The isolated yields of compounds **5** and **7** prepared in this reaction route with the use of the appropriate sodium salts were found to be 62%^1^ and 65%,^17^ respectively. Both complexes decompose without melting when heated (Table 1).

[Fe(urea)_6_](NO_3_)_2_(I_3_) (compound **8**) was prepared similarly by mixing a concentrated solution of iodine in sodium iodide (for each iron atom, six iodide and two iodine) with a concentrated solution of compound **1**. No complete metathesis was obtained with the formation of a triiodide compound, but a dinitrato-diiodoiodate (I_3_^–^) salt was formed.^12^ It might be attributed to the solubility of compound **8** being lower than that expected for [Fe(urea)_6_]I_3_.

A particular reaction route to prepare compounds **1** and **2** was found by Zhou et al., who used a solid-state reaction of urea with Fe(NO_3_)_3_·9H_2_O and FeCl_3_·6H_2_O (to prepare compounds **1** and **2**, respectively) in a 6:1 molar ratio in an agate mortar with intensive grinding for 6 h.^27^ Li et al. used the same procedure to prepare [Fe(urea)_6_]_2_(SO_4_)_3_ (compound **9**) from Fe_2_(SO_4_)_3_.^28^ The elemental analysis of complexes **1**–**8**, independent from the reaction routes, confirmed their Fe to urea stoichiometry as 1:6.

Compounds **2** and **3** are hydrated with 3 mol of water, as determined by vacuum drying over P_2_O_5_.^11,12^ Zhou et al. determined the melting points of compounds **1** and **2** as 136–139 and 178–180 °C,^27^ respectively. Russo et al.^15^ and Foca et al.^29,30^ found much lower melting points for compound **2**, 108 and 95–97 °C, respectively. The melting point differences given by these authors may be attributed to some water content in the sample prepared by Foca et al (**2CW** content in compound **2**) because **2CW** loses its crystal water around the temperature of the given melting point.^29,30^ Compound 2 is soluble in water and ethanol.^29,30^ For compounds **3** and **4**, Russo et al. determined the melting points, which were found to be 110^15^ and 202 °C,^16^ respectively. Compounds **1**–**4** were prepared by Carp et al.^31^ and Russo et al.^15,16^ according to eq 1. Their melting points are in Table 1. The magnetic measurements on these complexes showed the presence of high-spin iron(III) with 6.10,^31^ 5.56,^15^ 6.01,^15^ and 5.76 μ_B_^16^ experimental magnetic moment values for the nitrate, chloride, bromide, and perchlorate compounds, respectively. The molar conductivity (Λ) (in 10^–3^ mol·dm^–3^ concentration in a MeNO_2_ solution at 25 °C) of compound **4** was found to be 203.4 Ω^–1^·cm^2^·mol^–1^.^16^

Zhang studied the effect of the different Fe^III^ to urea mixing ratios (1:2, 1:3, 1:4, 1:5, 1:6, and 1:8) in the reaction of Fe(NO_3_)_3_·9H_2_O dissolved in 68% HNO_3_ and aqueous urea solutions, but the iron to urea ratios in the obtained products were found to be 1:6 in every case.^14^ The dark green crystalline compound **1** crystallized out in an 87% yield after the solution was concentrated at 70 °C to 1/3 of its original volume. The melting point was 164–165 °C (Table 1). Compound **1** readily dissolves in water, methanol, and DMSO, whereas it hardly dissolves in benzene, *n*-hexane, acetone, acetonitrile, and CCl_4_.^14^ Its molar conductivity (Λ) in water at 25 °C was 411.32 S·cm^2^·mol^–1^, indicating the 1:3 electrolyte nature of the complex. Zhang concluded that the nitrate ions in compound **1** are isolated and do not coordinate to the iron(III) center, confirming Barbieri’s assumption^11^ about the six-coordinate iron environment.^14^

A detailed kinetics study of the reaction between iron(III) nitrate and urea (resulting in compound **1**) was performed by measuring the thermal conductivities of iron(III) nitrate solutions in the absence or presence of urea. The formation reaction of compound 1 can be modeled with two different steps: In the first 10–40 s, the liquid film diffusion model can describe the reaction between iron nitrate and urea with the formation of dissolved [Fe(urea)_6_]NO_3_ without crystal formation. After 40 s, the formation reaction can be represented by the diffusion model of the formation of the urea-iron product layer.^32^ The thermal conductivities of the starting solutions were given as values between 25.68 and 11.63 × 10^4^ W·cm^–1^·K^–1^ (the high value is due to the low concentration of compound **1**).^32^

Maslowska first prepared the sulfate compound [Fe(urea)_6_]_2_(SO_4_)_3_ (compound **9**), according to eq 2, from aqueous solutions of compound **4** and Na_2_SO_4_.^18^ She determined the stability constants of compound **4** by the UV–vis method: β_1_ = 5.55, β_2_ = 9.55, β_3_ = 8.40, β_4_ = 5.80; β_5_ = 2.90, and β_6_ = 1.04; thus, the compound has higher stability than the [Fe(H_2_O)_6_]^3+^ complex cation.^18^ Maslowska et al. studied the complex stability of the [Fe(urea)_6_]^2+^ complex cation by polarography in the FeSO_4_–urea–NaClO_4_–H_2_O system. The stability constants were found as β_1_ = 2.6 ± 0.5, β_2_ = 8.0 ± 1.0, β_3_ = 7.0 ± 1.0, β_4_ = 15 ± 2, β_5_ = 5 ± 2, and β_6_ = 2 ± 2 for [Fe(urea)_6_]SO_4_; however, it was not possible to isolate it from the solution.^33^

Savinkina et al. studied the FeI_2_/urea/solvent (solvent = H_2_O) and [Fe(urea)_6_]I_2_–I_2_–H_2_O systems.^19^ The transparent yellow crystals of hexakis(urea)iron(II) iodide ([Fe(urea)_6_]I_2_, compound **10**) were formed at 0 °C in the FeI_2_–urea–H_2_O system. Compound **10** is very soluble in water, even at 0 °C.^19^ In the presence of excess I_2_ in the [Fe(urea)_6_]I_2_–I_2_–H_2_O system, [Fe(urea)_6_](I_3_)_2_ (compound **11**, polyiodide salt) was formed.^19^ If the system contained between 4.40 to 47.24 wt % [Fe(urea)_6_]I_2_ at 0 °C, an incongruently and slightly soluble diiodoiodate was formed as opaque, shiny black crystals ([Fe(urea)_6_](I_3_)_2_ compound **11**). IR and X-ray studies confirmed the formation of compounds **10** and **11**.^19^ The melting point and the pycnometric density of compound **10** were found to be ∼145 °C and 1.99 g·cm^–3^, respectively.^20^

Savinkina et al. and Kuz’mina et al. synthesized the [Fe(urea)_6_](I_3_)_3_ (compound **12**)^21,22^ and [Fe(urea)_6_]I_3_ (compound **13**)^34^ compounds according to the reaction shown in eq 1. The mixing of a hydroiodic acid solution containing dissolved urea and metallic iron results in compound **13**; however, only a slight excess of iodine already results in the formation of the polyiodide complex (compound **12**). Both solutions were slowly evaporated at room temperature, and shiny black (compound **12**) and yellow-orange columnar crystals (compound **13**) formed. The addition of urea to an aqueous solution of iron(II) decreases the redox potential of the Fe^III^ → Fe^II^ system. Thus, the oxidation power of the atmospheric oxygen will be enough to oxidize the iron(II) to iron(III).^19^ Single crystal measurements and magnetic susceptibility measurements^22,34^ proved the presence of iron(III) in compounds **12** and **13**. The magnetic moment values for compounds **12** and **13** were 6.30 and 5.69–5.99 μ_B_, respectively.^22,34^ Savinkina et al. measured the specific electrical conductivity (σ) and activation energy of electrical conductivity (*E*_a_) of the hexakis(urea)iron(III) diiodoiodate salt at 298 K (σ = 3.5 × 10^–5^ Ω^–1^·cm^–1^, *E*_a_ = 0.27 eV), respectively. After cooling, the electrical conductivity decreases significantly, and it becomes ∼10^–12^ Ω^–1^·cm^–1^ at 77 K.^21^ Savinkina et al. proposed that the charge transfer should occur predominantly along the complex’s anionic part due to the absence of a redox reaction of the complexing iron(III) ion (based on the magnetochemical measurements).^21^ It was strongly suggested that the structure must contain polyiodoiodate chains,^21^ which was confirmed by Kuz’mina et al. when they determined the structure of [Fe(urea)_6_](I_3_)_3_ and highly conductive (I_3_)^−^ channels.^34^

## 3 Crystallographic Features of Hexakis(urea)iron(II) and Hexakis(urea)iron(III) complexes

The available crystallographic parameters of known hexakis(urea)iron(III) complexes are summarized in Table 2.

**Table 2:** Summary of the Crystallographic Parameters of the Known Hexakis(urea)iron(III) Complexes

empirical formula	space group	unit cell dimensions	*Z*	*D* (g·cm^**-**^^3^)	*T* (K)	*V* (Å)^3^	*R*-factor (%)	ref (CCSD code)
[Fe(urea)_6_]Cl_3_	*R*3̅*c* (hexagonal)	*a* = 16.50 Å	6	1.482	295		13.0	(ZZZVTM)
		*c* = 14.90 Å						
	*R*3̅ (rhombohedral)	*a* = 16.75 Å	2					
		α = 100.0°						
[Fe(urea)_6_]Cl_3_·3 H_2_O	*R*3̅*c* (hexagonal)	*a* = 17.83 Å	6	1.480	295			(ZZZVRQ)
		*c* = 14.10 Å						
	*R*3̅ (rhombohedral)	*a* = 11.30 Å	2					
		α = 103.9°						
[Fe(urea)_6_](NO_3_)_3_	*A*2/*a* or *Aa*	*a* = 59.30 Å	92	1.680	295		2.90	(36)
		*b* = 18.59 Å						
		*c* = 52.70 Å						
		β = 105.3°						
	*C*2/*c* or *Cc* (substructure)	*a* = 11.25 Å						
		*b* = 18.59 Å						
		*c* = 12.49 Å						
		β = 111.2°						
[Fe(urea)_6_](I_3_)_3_	*P*6_1_	*a* = 12.072(4) Å	6	2.961	295	5243(3)	3.08	(MANJIU)
		*c* = 41.54(2) Å						
[Fe(urea)_6_]I_3_^a^	*R*3̅	*a* = 17.626(9) Å	6	1.780	295	3750.6	7.28	(WITQEV)
		*c* = 13.940(8) Å						
[Fe(urea)_6_](MnO_4_)_3_	*P*2_1_/*c*	*a* = 13.701(0) Å	4	1.981	100	2592.2	4.9	(KELPIE)
		*b* = 10.008(0) Å						
		*c* = 11.413(0) Å						
		β = 112.99(0)°						
[Fe(urea)_6_]_2_(S_2_O_8_)_3_	*P*1̅	*a* = 9.9125(7) Å	2	1.874	100	1248.4	13.0	(XEVKIW)
		*b* = 11.916(8) Å						
		*c* = 12.919(1) Å						
		α = 63.174(8)°						
		β = 88.604(9)°						
		γ = 68.476(8)°						

a The [Fe(urea)_6_]I_3_ was mistakenly uploaded to CCSD database as [Fe(urea)_6_]I_2_; however, the article^22^ clearly described an iron(III) complex.

**Figure 1:**
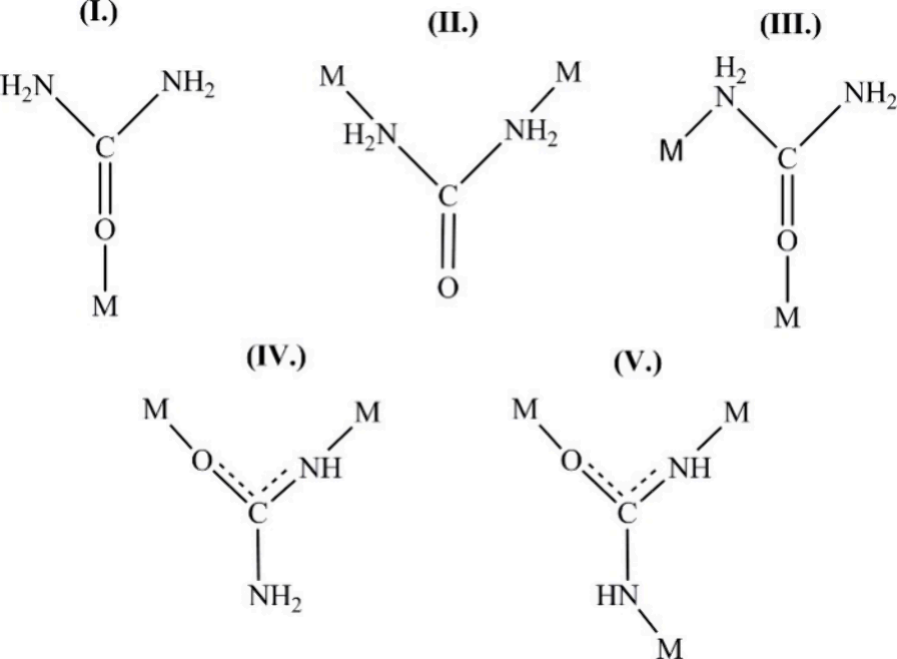
Possible coordination forms of the urea molecule. Reprinted with permission from ref (37). Copyright 2007 Labrini Drakopoulou et al.

The urea molecules have different possibilities (via O or N) for coordinating with the central metal ion in mono- or multidentate^13,37^ coordination modes (Figure 1).

**Figure 2:**
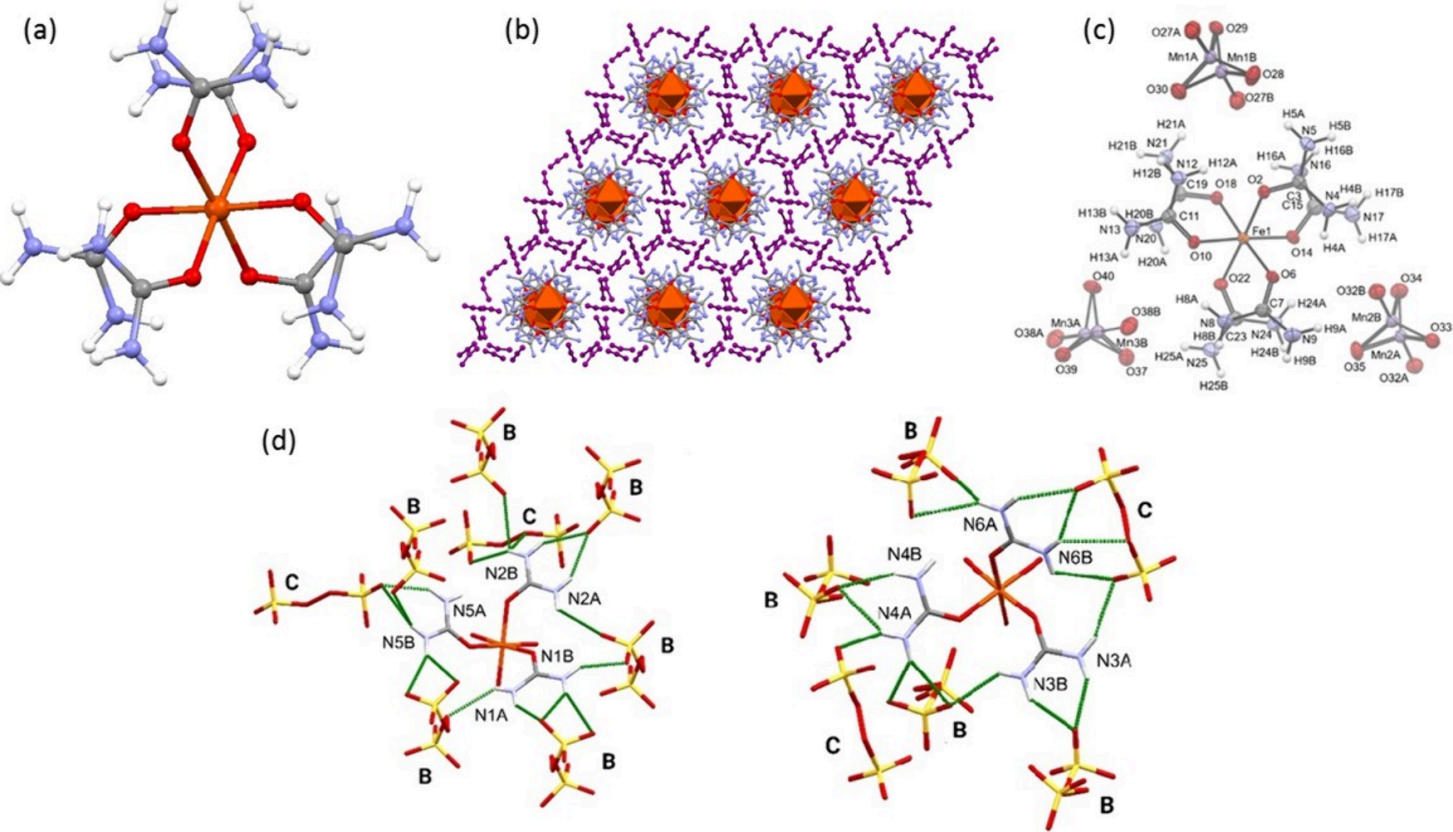
Crystal structures of (a) the [Fe(urea)_6_]^3+^ ion,^1^ (b) compound **12**,^34^ (c) compound **5**,^1^ and (d) compound **7** (showing the two kinds of anions).^17^ (a and c) Reprinted with permission from ref (1). Copyright 2022 American Chemical Society. (d) Reprinted with permission from ref (17). Copyright 2022 Béres et al.

However, in all the cases evaluated by us, the six urea ligands coordinate with the central Fe^III^ ion as monodentate ligands via their oxygen atom (*k*^1^-O), resulting in an octahedral arrangement around the central iron ions (Figure 2a).

Okaya^35^ performed the first crystallographic study on the members of this compound family. The crystallographic parameters of hexakis(urea)iron(III) trichloride and its trihydrate (compounds **2** and **2CW**, respectively)^35^ were deduced from Patterson and density projections; in the case of compound **2** and compound **2CW**, Okaya et al. determined the parameters based on the projections along the trigonal and hexagonal axes, respectively, in the *a*- and *c*-directions (Table 2).^35^ Hexagonal and rhombohedral settings were used, and the yellow crystals belonged to the *R*3̅c and *R*3 space groups, respectively.^35^ Due to the rudimentary crystallographic techniques, an approximate position relation could be determined. If the iron was in the (0, 0, 0) position in a Descartes coordinate system, then the Cl^–^ should be in the (1/3, 0, 1/4) position.^35^ Okaya et al. proved that the urea ligands coordinated via their oxygen atom and were positioned in an octahedral arrangement.^35^ Durski et al. and Aliyeva et al. studied compounds **1** and **2**, respectively, with powder X-ray diffraction analysis.^36,38^ Durski et al. determined the unit cell parameters of hexakis(urea)iron(III) trinitrate (compound **1**) with the Weissenberg and the reverse lattice photography method^36^ (Table 2). Durski et al. proposed that compound **1** belongs to the monoclinic crystal system and showed features characteristic of a superlattice.^36^ Later, detailed single crystal X-ray diffraction measurements of compounds **5**,^1^**7**,^17^**12**,^34^ and **13**(22) were performed. The orientation of urea ligands in the complex is similar in all cases. Still, the rotation directions at the two sides of the cations are different (giving a propeller-like shape) (Figure 2a).

The propeller-like orientation causes a helical chirality. The Fe–O bond lengths and O–Fe–O angles depend on the nature of the counterions and positions within the octahedral and vary between 1.960 and 2.020 Å and between 85.3– 93.5° and 174.2–177.9°, respectively. The C=O and C–N bond lengths depend on the coordination strength of the urea ligand to iron. Accordingly, these vary for these hexakis(urea)iron(III) salts between 1.220–1.282 and 1.270–1.380 Å, respectively. The shortest Fe–Fe distances varied between 6.433 and 6.976 Å.^1,17,22,34^

The hexakis(urea)iron compounds [Fe(Urea)_6_](I_3_)_3_ (compound **12**)^34^ and [Fe(Urea)_6_]I_3_ (compound **13**)^22^ formed in the Fe^II^/Fe^III^–I^–^/I_2_/I_3_^–^–urea system were studied by Kuz’mina et al. (Table 2). The shiny blackish crystals of compound **12** belonged to the hexagonal crystal system. The diiodoiodate anions are located between the complex cations in a zigzag-like manner, forming chains through the whole unit cell and forming hexagons around the complex cations (Figure 2b). The distances of I_3_^–^ ions vary between 3.769 and 4.021 Å, and intermolecular interactions between the urea ligands and polyiodoiodate anions (N–H···I hydrogen bonds) stabilize the structure.^34^ Compound **13** crystallizes in the form of yellow-orange columnar crystals and belongs to the trigonal *R*3̅ system. It has a stack-like structure: stacks of I^–^ are oriented along the *z*-axis, forming channels with a hexagon-shaped cross-section around the *z*-axis in the center of the channel. The complex cations can be found inside these channels along the same axis. There is an extended N–H···I type hydrogen bond system between the [Fe(urea)_6_]^3+^ and I^–^ ions.^22^

Béres et al. studied the structure and hydrogen bond features of two hexakis(urea)iron(III) compounds with oxidizing anions, hexakis(urea)iron(III) permanganate (compound **5**(1)), and hexakis(urea)iron(III) peroxydisulfate (compound **7**(17)) (Table 2). The cryo-DSC studies on these compounds showed that no phase transformations occurred between −140 K and their decomposition temperatures.^1,17^

The dark purple permanganate compound (compound **5**) shows unique structural features. Its structure shows notable pseudosymmetry since the complex cation and anions fit *R*3̅*c* space group symmetry. The three anions (that were found in the asymmetric unit) are in disordered orientations, facing up (anion A) or down (anion B), and their distributions are 94.19% and 5.81%, respectively (Figure 2c). Moreover, the permanganate anions form zigzag-like channels between the complex cations (just like in the case of compound **9**). The smallest Fe–Fe distances between the Fe^III^ ions were 6.667 and 7.037 Å, and there were potential solvent-accessible voids (about 0.8% of the whole structure) between every two [Fe(Urea)_6_]^3+^ ions.^1^ An extended inter- and intramolecular hydrogen bonding system between the complex cations and anions (N–H···O–Mn) and inside the complex cation (N–H···O=C) stabilizes the whole structure. The hydrogen bonding system also shows pseudosymmetry, since not all intermolecular hydrogen bridges were present in every ligand.^1^

The persulfate compound (compound **7**) forms light blue triclinic blocks.^17^ Its structure consists of two kinds of S_2_O_8_^2–^ ions (Figure 2d, B and C) in the asymmetric unit, and the two kinds of anions are involved in two different hydrogen bonding systems (both their the number and position are different) (Figure 2d).^17^ The two halves of the anion C (O_3_S–O−) form hydrogen bonds symmetrically with the same number of hexakis(urea)iron(III) complex cations, whereas the two halves of the anion B have asymmetric hydrogen bond networks; one half of the O_3_S–O– unit forms 11 hydrogen bonds, while the other half forms 9 hydrogen bonds.^17^

## 4 Spectroscopic Characters of the Hexakis(urea)iron(III) Complexes

### 4.1 Vibrational Spectroscopy (Ir and Raman Spectroscopy)

**Figure 3:**
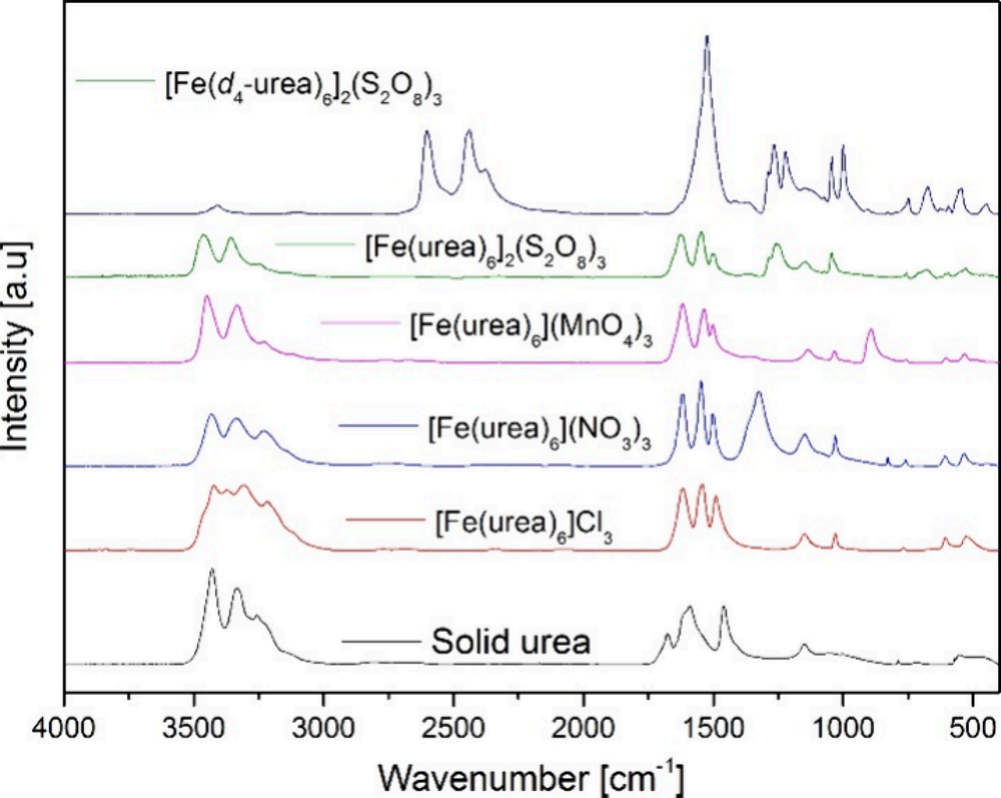
Analytical-range IR spectra of solid urea and different hexakis(urea)iron(III) complexes. Reprinted with permission from ref (1). Copyright 2022 American Chemical Society. Reprinted with permission from ref (17). Copyright 2022 Béres et al.

The IR and Raman spectroscopy methods are the most widely used to determine the coordination mode (N or O coordination) of urea to iron centers in compounds with no known crystal structures. When C=O coordination occurs, as typical for hexakis(urea)iron compounds with known structures, the strength of the C=O bond decreases, whereas that of the C–N bond increases. Accordingly, the peak of the C=O stretching mode gets shifted to a lower wavenumber, while the peak of the C–N mode is moved to a higher wavenumber compared to pure solid urea (Figure 3.).^13,40−43^ Most of the research reports used the ν(C=O) and ν(C–N) modes to confirm the formation of hexakis(urea)iron complexes, since these modes have characteristic band positions for the complexes.^14−16,29−31^ In the case of hexakis(urea)iron(II/III) complexes, the ν(C=O) symmetric stretching mode is expected between 1580 and 1500 cm^–1^ in the form of an intense peak (Figure 3).^13,40−43^ The ν(C–N) antisymmetric and symmetric stretching modes are expected between 1500 and 1450 cm^–1^ and 1050 and 1010 cm^–1^, respectively. However, they usually combine with the ρ(N–H), δ(N–H), and δ(C=O) modes (Figure 3).^13,40−43^ Therefore, these pure modes are hard to isolate even with low-temperature Raman measurements.^1,17^ The deuteration of compound **7**, however, allowed us to decompose the overlapped ν(C=O) and ν(C–N) modes around 1500 cm^–1^ because the N–H/N–D bond transformation has a greater influence on the positions of the C–N bands than those of the C=O bands (Figure 3).^17^ The NCO and NCN deformation modes were around 650 and 540 cm^–1^, respectively (Figure 3),^13^ whereas the ν(Fe–O) bands were between 310–300 cm^–1^. The lattice vibrations around 240–210 cm^–1^ were assigned in the far-IR region.^42,43^

The bands of the N–H vibrational modes of hexakis(urea)iron salts are located at three parts of their vibrational spectra.^1^ The symmetric and antisymmetric stretching modes of −NH_2_ groups appear as a set of intense peaks together with some low-intensity combination/overtone bands in the spectral range from 3500 to 3200 cm^–1^ (Figure 3).^1,13−18,29−31,40−44^ Sulaimankulov et al.^40^ showed that the narrow peaks of the τ(NH_2_) band at 540 cm^–1^ may be attributed to the presence of hydrogen bonds, which form in electron-donor solvents and the solid state as well,^13,39^ and the deformation and rocking modes of the NH_2_ groups appear around 1650 and 1190–1100 cm^–1^, respectively(Figure 3).^1,13−18,29−31,40−44^ Deuteration resulted in an excellent tool to distinguish these bands from other vibrational modes due to the shifting of ND_2_ modes to lower wavenumber, with ν(NH)/ν(ND) about 1.35.^17^

The Raman spectra of the hexakis(urea)iron(III) tris(diiodoiodate) salt (compound **12**)^43^ showed two intense (ν_2_ and ν_3_) and one weak (ν_1_) Raman modes of the I_3_^–^ ion at 147, 113, and 93 cm^–1^. Since these anion bands are located in the far-IR range, the cation modes can be assigned easily without overlapping with anion modes. The decreased symmetry of the centrosymmetric compound **12** results in the activation of Raman inactive ν_1_ and ν_3_ modes of the anion, as found in the IR spectrum of the permanganate compound (compound **5**). This led to otherwise IR-inactive symmetric stretching and deformation modes appearing in the spectrum.^1^ The stretching modes are at 909 and 899 cm^–1^ (ν_as_(Mn–O), the highest intensity peak) and at 838 cm^–1^ (ν_s_(Mn–O), the lowest intensity peak), and the two deformation modes are located at 375 (δ_as_ (Mn–O), broad band) and at 307 cm^–1^ (δ_as_ (Mn–O)) (Figure 3).^1^ The low-intensity peak of ν_s_ in the IR spectrum is the most intense one (at 840 cm^–1^) in the Raman spectrum, whereas the least intense Raman peak is a doublet at 922 and 905 cm^–1^ (ν_as_(Mn–O)) (Figure 3).^1^

Sulaimankulov et al. found that the IR spectra of compounds **1** and **2** in *d*_4_-methanol were similar to those determined by Penland et al. in KBr pastille.^39,40^ This was attributed to the fact that there were large aggregates of the molecules in the solution, which was proved by EPR measurements. The EPR spectrum of the chloride compound (compound **2**) in *d*_4_-methanol and the solid state exhibited a singlet and a triplet peak with *g*_eff_ = 2, respectively. This showed that the iron(III) must be in a different ligand field in the solution.^40^

### 4.2 UV–vis Spectroscopy

Jørgensen determined the electronic transitions of the [Fe(urea)_6_]^3+^ complex cation^45,46^ as ^4^Γ_4_(G) ← 6∑ = 800 nm, ^4^Γ_5_(G)← 6∑ = 584 nm, ^4^Γ_1_(G) ← 6∑ = 433 nm, and ^4^Γ_3_(G) ← 6∑ = 427 nm. Holt et al. measured the UV–vis spectra of hexakis(urea)iron(III) perchlorate at both 298 and 20 K temperatures and with different polarizations (σ and π).^47^ They propose that due to the octahedral Fe^III^, the d^5^ (t_2g_(3), e_g_(2) configuration) system that results in a 6A_1_ ground electronic state, the transition will be spin-forbidden.^42^ Two bands were observed in polarizations between 1000 and 500 nm at 298 K. The peaks belonged to the ^4^*T*_1_ ← ^6^*A*_1_ and ^4^*T*_2_ ← ^6^*A*_1_ transitions appearing at 833 and 629 nm with π-polarization and at 851 and 599 nm with σ-polarization, respectively, together with an additional shoulder at 794 nm.^47^ There were two intense peak systems (ε > 3.1) found belonging to the ^4^*E* ← ^6^*A*_1_ and ^4^*A*_1_ ← ^6^*A*_1_ transitions in both polarizations (435 and 429 nm) with four and one shoulders between 430 and 418 and 428 nm at the π and σ polarizations, respectively.^47^ Holt et al. found a well-shaped peak around 390 nm and a shoulder with σ- and π-polarizations belonging to an electric dipole transition (^4^*T*_2_(D) ← ^6^*A*_1_).^47^ These assignations were confirmed by Jørgensen’s measurements^45,46^ and Cotton’s theoretical calculations.^48^

Carp et al. measured the UV–vis spectra of hexakis(urea)iron(III) nitrate.^31^ Two broad, low-intensity peaks with shoulders ∼600 and ∼850 nm, belonging to the ^4^*T*_2g_(G) ← ^6^*A*_1_ transition and the ^4^*T*_1g_(G) ← ^6^*A*_1_ transition, respectively. The ^4^*T*_2g_(G) ← ^6^*A*_1_ and ^4^*A*_g_, ^4^*E*_g_(G) ← ^6^*A*_1_ transitions of compound **7** were observed at 590 (very weak and broad band) and 432 nm, respectively, and both transitions are due to the degeneration of the octahedral symmetry of the [Fe(urea)_6_]^3+^ ion.^17^ Due to the purple color of compound **5,** none of these transitions could be assigned.^1^

### 4.3 X-ray Spectroscopy

The electronic structures of some hexakis(urea)iron(II/III) complexes were studied in detail with X-ray technique. Jørgensen et al. studied the X-ray photoelectron spectra of [Fe(urea)_6_](ClO_4_)_3_ (compound **4**), and the following peaks were assigned: I(2p_1/2_) at 735 and 731.7 eV; I(2p_3/2_) at 726 and 717.8 eV; I(3p) at 63 and 62 eV; I(3_d_) at 10.2 eV for iron(III); and I(2p_3/2_) for chlorine in ClO_4_^–^.^49^ Narbutt et al. studied the Kβ_1_ line of chlorine in hexakis(urea)iron(III) chloride, since this line corresponds to the chlorine 3p electronic shell.^50^ The position of this peak of compound **2** is at 2815 eV, but the different shape of this line observed at the same position for NaCl shows that the chlorine bonding in the crystal structure of compound **2** is not purely ionic. Mixing the chloride 3p and cation orbitals may lead to the hydrogen bonding between the chlorine and the complex cation ligands.^50^ Tamaki et al. concluded that the shift (+0.5 eV) of the Kβ_1,3_ line of iron(III) in hexakis(urea)iron(III) chloride trihydrate is mainly due to the influence of the ligand on the d-shell of iron(III).^51,52^ This finding agrees well with the later Mössbauer results of Russo et al. and Béres et al. about the influence of the anions on the d-shell of the central metal ion in hexakis(urea)iron(III) complexes.^1,15−17,53^

Tami et al. studied the K edge 1s → 3d pre-edge features of the perchlorate compound (compound **4**) with X-ray absorption spectroscopy (XAS) and extended X-ray absorption fine structure (EXAFS) methods. Since the cation of compound **4** has *O*_h_ geometry (thus it is centrosymmetric), the only the transition allowed is the electric quadrupole transition, (*t*_2g_)^3^(*e*_g_)^2^ (^5^*A*_1g_) → (*t*_2g_)^2^(*e*_g_)^2^ (^5^*T*_2g_) or (*t*_2g_)^3^(*e*_g_)^2^ (^5^*A*_1g_) → (*t*_2g_)^3^(*e*_g_)^1^ (^5^*E*_g_). These two peaks appear at 7113 and 7114 eV on the XAS spectra of compound **4**.^54^

### 4.4 Mössbauer Spectroscopy

**Figure 4:**
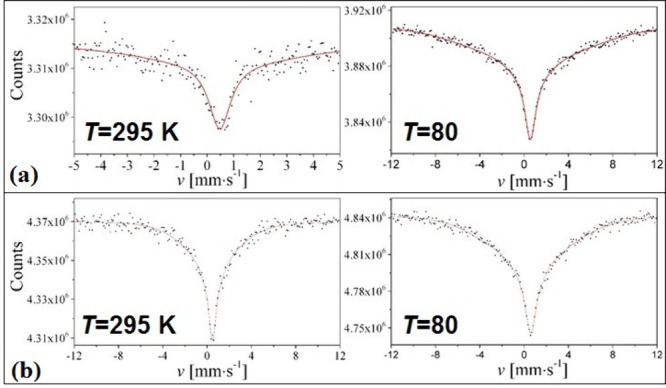
Mössbauer spectra of (a) compound **5** and (b) compound **7** at different temperatures. (a) Reprinted with permission from ref (1). Copyright 2022 American Chemical Society. (b) Reprinted with permission from ref (17). Copyright 2022 Béres et al.

The Mössbauer spectra of hexakis(urea)iron(III) chloride, bromide,^15,53^ perchlorate,^15,53^ nitrate,^54^ permanganate,^1^ and peroxydisulfate^17^ were measured at various temperatures between 80 and 295 K (Table 3). All studied compounds proved to be high-spin iron(III) complexes. However, the spectral shape is always a broadened Lorentizan singlet (paramagnetic spin relaxation line shape), which is difficult to evaluate (for example, compounds **5** and **7** in Figure 4).

**Table 3:** Mössbauer Parameters of Hexakis(urea)iron(III) complexes with Different Anions (X)^a^

	X = Cl^–^		X = Br^–^		X = ClO_4_^–^		X = NO_3_^–^		X = MnO_4_^–^b		X = S_2_O_8_^2–^b	
*T* (K)	δ	Γ	δ	Γ	δ	Γ	δ	Γ	δ	Γ	δ	Γ
80	0.58	0.87	0.57	0.90	0.33	0.89	no data were given in ref (55)		0.501	0.875	0.542	0.996
100	0.58	0.88	0.54	1.04	0.34	0.88			-c	-c	-d	-d
150	0.57	0.88	0.53	0.80	0.37	0.89			-c	-c	-d	-d
200	0.59	0.87	0.41	0.89	0.36	0.90			-c	-c	-d	-d
295	0.60	0.90			0.35	0.90			0.412	0.626	0.416	0.608

a δ, isomer shift relative to α-iron; Γ, full width at half-weight. All parameters are given in mm·s^–1^.

b The Bluem–Tjon two-state relaxation model was used to evaluate the spectra.^1,12^

c No data were given in ref (1).

d No data were presented in ref (17).

Galeazzi et al. and Béres et al. used IEHM and DFT calculations, respectively, to prove the correctness of their evaluation, which showed good agreement between the experimental and calculated parameters.^1,17,53^

Russo et al. evaluated the Mössbauer spectra with a single Lorentizan line. However, the curve did not fit well on the two sides of the spectra. Yamauchi et al. and Béres et al. used a Bluem–Tjon two-spin state relaxation model,^1,17,54^ which gave a better fit. However, the Mössbauer parameters (like the spin relaxation frequency of the electric field gradient, *V*_*zz*_) correlate highly with the isomer shift (δ). The broad lines are due to the spin–spin and spin–lattice interactions.^1,17^ The full widths at half-height (Γ) did not change upon cooling for the compounds **1**–**4** because the spin–spin interaction dominates these materials.^15,16,55^ However, the Γ values were changed upon cooling for compounds **5** and **7**; thus, spin–spin and spin–lattice interactions may contribute to the line distortion.^1,17^ Yamauchi et al. and Béres et al. proposed that the Fe^3+^–Fe^3+^ distance and the crystal lattice’s rigidity strongly influence the line shape.^1,17,55^ If the Fe^3+^–Fe^3+^ distance is between 6 and 12 Å, the line shape shows paramagnetic spin relaxation, but over this distance slightly magnetically split spectra can be seen.^55^ Moreover, Russo et al. and Béres et al. proposed that the change of δ in the case of different anions is due to the influence of the anion on the s-electron density at the iron(III) nucleus. Since the Cl^–^-and Br^–^-containing complexes have higher δ parameters than the other three derivatives (X = ClO_4_^–^, MnO_4_^–^, and S_2_O_8_^2–^) (Table 3), this means that the hydrogen bond networks also play a role in the decrease of the d-shell electron density of Fe^III^ ion resulting in lower isomer shift.^1,15−17,53^

### 4.5 EPR Measurements

Sulaimankulov et al. measured the EPR spectra of hexakis(urea)iron(III) chloride both in the solid state and dissolved in *d*_4_-methanol, concluding that the iron(III) is in a different ligand field in solution and the solid state.^39,40^ Cotton et al. recorded the EPR spectra of hexakis(urea)iron(III) nitrate, chloride, and perchlorate in two different magnetic fields (9.3 (X-band) and 36 GHz (Q-band)).^44^ X-band resonance can be found in this magnetic field region, whereas the Q-band was found at *g*_eff_ = 2. The two spin-Hamiltonian parameters (*D* and λ) were determined for all three complexes ([Fe(urea)_6_]Cl_3_, *D* = 0.13 cm^–1^ and λ = 0.067; [Fe(urea)_6_]X_3_ (X = NO_3_^–^, ClO_4_^–^), *D* = 0.070 cm^–1^ and λ = 0.067), shwoing a zero-field split in all three complexes.^44^ The differences between the *D* and λ values indicate that the anions influence the ferric ion, which was previously assumed by Narbutt et al.^50^ based on their X-ray fluorescence analysis.

## 5 Thermal Behavior of Hexakis(urea)iron(III) Complexes

The complex cations in the hexakis(urea)iron(III) salts have three positive charges and are surrounded by many negatively charged anionic particles. The anions interact with the polarized N–H bonds of urea ligands and form extended hydrogen bond systems. This structural feature strengthens the lattice energy of the complexes. Consequently, the melting points of these complexes are expected to be high (see Table 1). Thermal decomposition of these compounds may take place either in solid phase or melt in two possible routes:

1. The decomposition starts with a simple ligand loss and consecutive decomposition of intermediates in the solid or molten phase. The ligand loss is always an endothermic process. The next decomposition steps may involve the reactions of urea (e.g., oxidation in air) or other decomposition intermediates, which can change the reaction into an exothermic process. This decomposition route was observed in the case of hexakis(urea)iron(III) nitrate (compound **1**) and hexakis(urea)chloride (compound **2**).^26,32,56,57^
2. The other decomposition reaction route occurs when the ligand and anion interaction occurs in the solid phase and before urea ligand loss. These redox reactions between the ligand and anion always generate heat; thus, these reactions start with an exothermic character even in an inert atmosphere. The hexakis(urea)iron(III) complexes having strongly oxidizing cations, for example, hexakis(urea)iron(III) permanganate (compound **5**) and hexakis(urea)iron(III) peroxydisulfate (compound **7**) belong to this group.^1,17^

The solid phase quasi-intramolecular redox reactions generally occur at low temperatures (between 50–140 °C), and the reactions are exothermic; due to the solid-phase environment and low temperature, these redox reactions result in a disrupted lattice product (amorphous materials), which can crystallize in controlled size with postannealing.^1,17,58−67^ The oxidation power and reducing ability of the ligands in the solid phase are not equal to those parameters found in aqueous solutions. Accordingly, the urea as a reducing ligand will cause special effects in the gaseous environment, and various valence states of the metals may appear in the solid mixed oxides.

**Figure 5:**
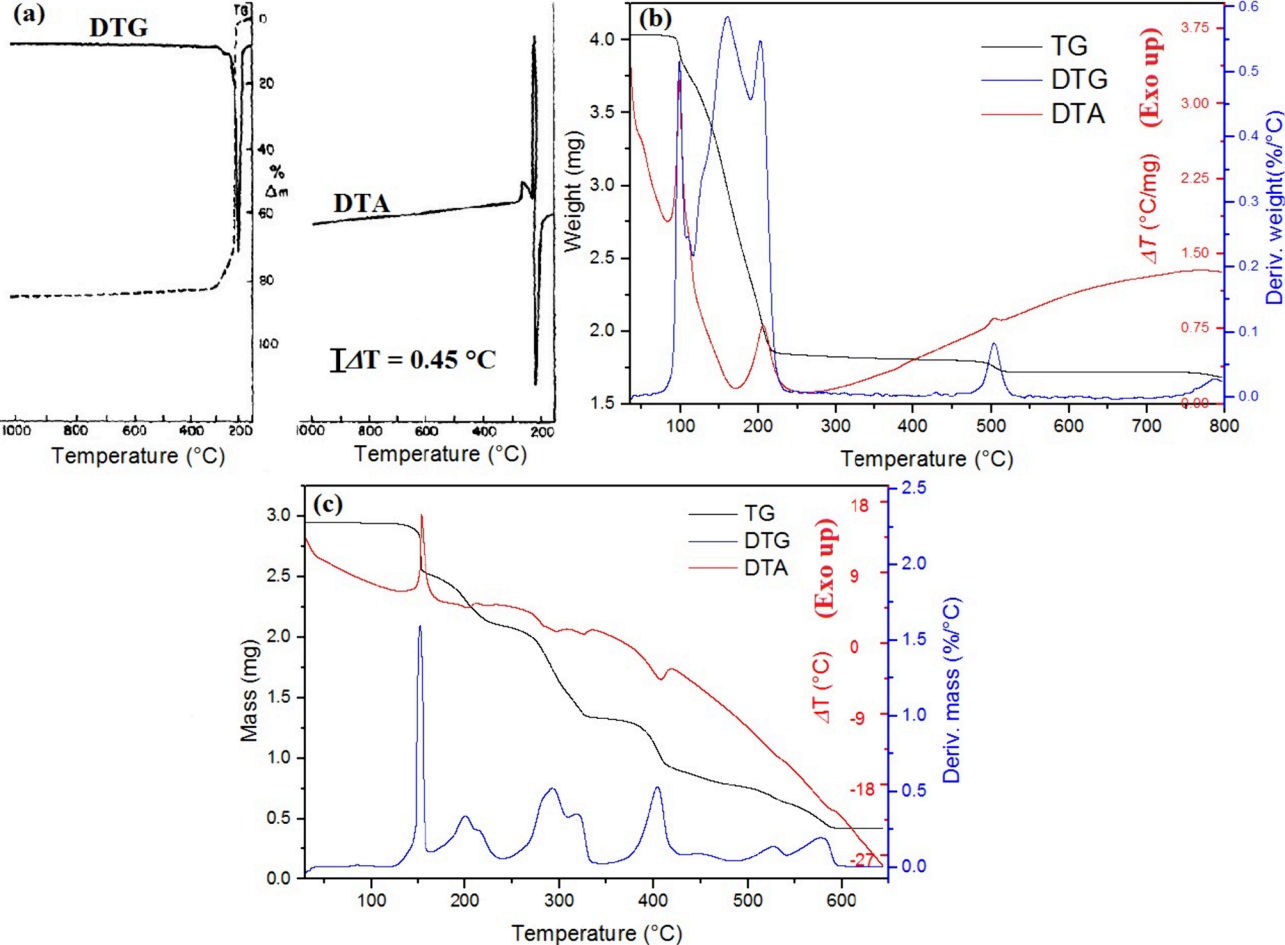
TG-DTG-DTA curves of (a) compound **1**, (b) **5**, and (c) **7** under an inert atmosphere. (a) Reproduced with permission from ref (56). Copyright 1984 Published by Elsevier B.V. (b) Reprinted with permission from ref (1). Copyright 2022 American Chemical Society. (c) Reprinted with permission from ref (17). Copyright 2022 Béres et al.

The comparison of the TG-DTG-DTA curves of the hexakis(urea)iron(III) nitrate, permanganate, and persulfate complexes can be seen in Figure 5.^1,17,56^

The amorphous iron-containing oxide materials are important, e.g., as catalysts in various industrial processes. Therefore, the thermal decomposition reaction routes of the hexakis(urea)iron compounds for preparing these useful mixed oxide products need attention. Information on the decomposition of some very important hexakis(urea)iron(III) complexes is collected in the following sections.

### 5.1 Hexakis(urea)iron(III) Trinitrate: Compound **1**

**Figure 6:**
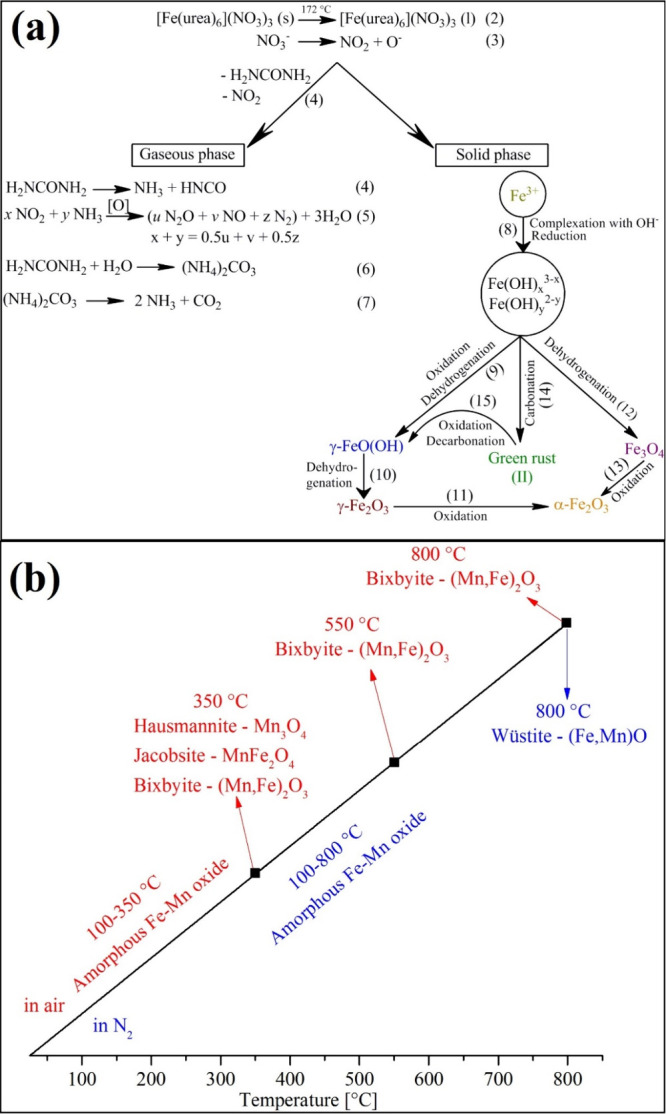
Thermal decomposition root of (a) compound **1** and (b) final products of compound **5**. (a) Reprinted with permission from ref (31). Copyright 2002 Elsevier Science B.V. (b) Reprinted with permission from ref (1). Copyright 2022 American Chemical Society.

Lupin et al. studied the hexakis(urea)iron(III) nitrate with TG, DTG, and DTA methods (both in air and argon atmosphere) up to 1000 °C (with 5 °C·min^–1^ speed) and characterized the residue of the decomposition by the powder X-ray diffraction method.^56^ Compound **1** decomposes in two steps in an argon atmosphere: an endothermic reaction starts between 181 and 219 °C (weight loss: 75.0% (calc. 75.3%)) and the second exothermic reaction occurs between 219 and 388 °C (weight loss: 9.9% (calc. 11.8%)) (Figure 5a).^56^ Only a single decomposition step in air was found between 174 and 251 °C. However, this step is separated into two parts: intense endothermic and intense exothermic peaks appear in the DSC curves at 189 and 201 °C, respectively.^56^ The endothermic peaks are attributed to the melting of compound **1** and urea ligand loss. Zhang et al. gave 136–139 °C as the melting point.^14^ The exothermic reaction is attributed to the redox reaction between the urea and nitric oxides in the melt phase.^56^ Based on the data, the following decomposition root (in inert atm.) was proposed by Lupin et al. (eqs 3 and 4):^56^
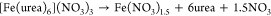
3
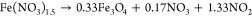
4Between 300 and 400 °C, a low-intensity exothermic peak system can be seen in both atmospheres (Figure 6a), which the authors did not analyze. The final products at the end of the heat treatment are Fe_2_O_3_ and Fe_3_O_4_ in oxidative and inert atmospheres, respectively.^56^ Akiyoshi et al. found the same decomposition temperature range and weight loss for compound **1** (in argon).^68^ They calculated the activation energy with the help of the DTG-based weight loss and a first-order equation as 160.4 kJ·mol^–1^.^68^ Furthermore, the mixture of compound **1** with KClO_4_ or KBrO_3_ was also measured. The decomposition reactions were single-step processes with intense exothermic character (peak maxima was at 175 °C), similar to the decomposition in air. The weight loss was found to be less, around 64%.^68^ Akiyoshi et al. studied the formation of the gases during these complex decomposition reactions: a large amount of CO_2_, N_2_, and NH_3_ formed, while a small amount of CO, NO, and NO_2_ formed during the procedure (N_2_O was not found), and Fe(NO_3_)_2_ formed instead of Fe_3_O_4_.^68^

Carp et al. studied the decomposition reaction of compound **1** in inert and oxidative atmospheres with a GC-TG-DTG-DTA method.^31^ They characterized the composition of the formed gas mixture with a MS technique up to 800 °C (with 1.5 °C·min^–1^ speed).^31^ Slightly less weight loss was found than that observed by Lupin et al. (83.9% and 83.7% inert and air, respectively). Moreover, after the first endothermic reaction (recognized by Lupin et al.^56^), the authors found three decomposition steps between 155 and 250 °C based on the DTG curve. The first was a rather exothermic reaction with about 75% weight loss. During this decomposition reaction, H_2_, H_2_O, CO_2_, NH_3_, N_2_, NO_2_, and HNCO formed^31^ (similar to the Akiyoshi et al. study^68^). The second and third steps (about 9% summa weight loss) were endothermic and exothermic, respectively, and the formation of H_2_O and CO_2_ was found. The decomposition residues (based on MS data and PXRD, Mössbauer, and UV–vis measurements) were as follows with the increase of temperature: Fe(OH)_*x*_ (X = 2 and 3) → FeO(OH) and γ-Fe_2_O_3_. The phase transition γ-Fe_2_O_3_→ α-Fe_2_O_3_ at 430 °C resulted in the final decomposition product.^31^ The proposed thermal decomposition route is summarized in Figure 6a. Zhao et al., who used TG-DSC,^69,70^ and Asuha et al., who used the TG-DTA technique,^71^ concluded the same results. Furthermore, Zhao et al. proposed the same decomposition step for the first stage in air as Lupin et al. in an inert atmosphere.^56^ However, the second step was described by eq 5.^69,70^
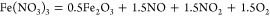
5

### 5.2 Hexakis(urea)iron(III) Permanganate: Compound **5**

**Figure 7:**
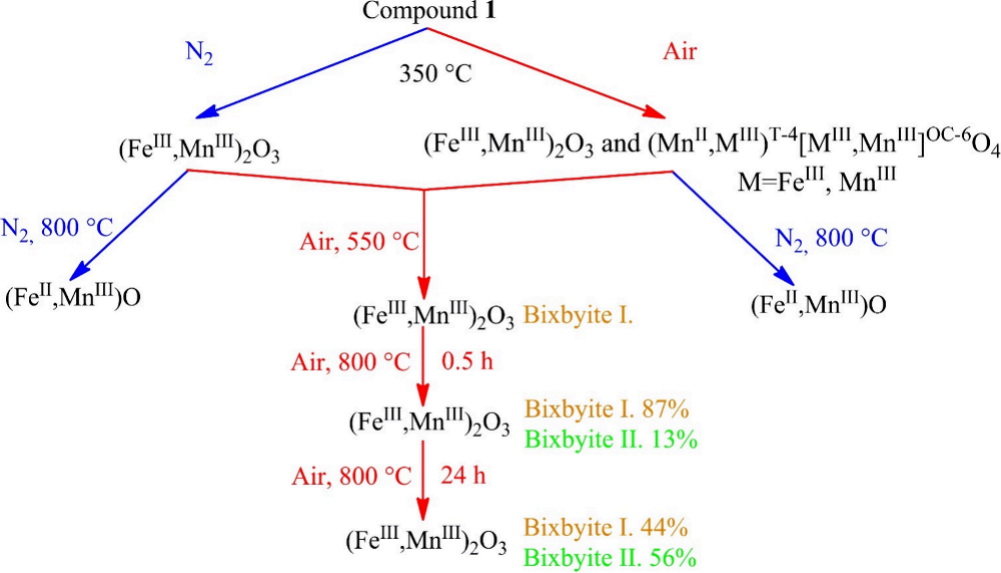
Thermal decomposition root of compound **5** in the inert, oxidative, and switched (from oxidative to inert) atmospheres. Reprinted with permission from ref (1). Copyright 2022 American Chemical Society.

Béres et al. studied the thermal decomposition of compound **5** with TG-MS and DSC methods.^1^ The total weight loss until 800 °C was 41.0% and 37.3% in air and an inert atmosphere, respectively.^1^ In both atmospheres, the decompositions were multistep processes and started with an intense (explosion-like) exothermic reaction at 94 °C. The presence of oxygen did not have an essential role in the initiation of the decomposition. However, the heat of the reaction was found to be 446.31 and 341.1 J·g^–1^ in the oxidative and inert atmospheres, respectively. Thus, the aerial oxygen took part in the oxidation of urea ligands. The decomposition reaction temperature was lower than the ligand loss temperature of compound **1**,^1^ and the reaction started exothermally. Thus, a heat-induced solid-phase quasi-intramolecular redox reaction occurred between the urea ligand and permanganate anion. It occurred inside the lattice structure, which was confirmed by the scanning electron microscopy measurements showing the unaltered morphology of the starting material.^1^ The decomposition of the complex in the inert atmosphere takes place in seven steps, whereas in the air it takes five separate steps (based on the DTG curve). The final products of the inert and the oxidative atmosphere heat treatment are summarized in Figures 6b and 7.^1^

The first decomposition step (between 94 and 120 °C) in both atmospheres produces H_2_O, CO_2_, NO/N_2_, and NO (urea oxidation products), while above 120 °C (second step) NH_3_, CO_2_, H_2_O, HNCO (urea decomposition products) formed.^1^ The IR spectrum of the first decomposition intermediate showed no MnO_4_^–^ peak, but in the residue formed above 120 °C urea, biuret, and isocyanate bands were detected, which were included in the decomposition reaction of urea (via water or ammonia elimination). The same urea decomposition products were observed in the decomposition of compound **1**.^1,31^

### 5.3 Hexakis(urea)iron(III) Peroxidisulfate: Compound **7**

Béres et al. studied the thermal behavior of compound **7** in detail under inert (nitrogen) and oxidative atmospheres, with TG-MS and DSC techniques up to 800 °C.^17^ During the oxidative atmosphere heat treatment, the final weight loss was 85%, while in the inert atmosphere it was 75%; however, in both cases the final product was hematite (α-Fe_2_O_3_). Similar to compound **5**, the decomposition of the complex started at 130 °C, below the ligand loss/urea melting point (*T*_mp_ = 135 °C) temperature, and the decomposition had an exothermic character in both atmospheres. The *ΔH*_r_ values were 386.45 and 375.93 J·mol^–1^ in inert and oxidative atmospheres, respectively. The formation of gaseous urea oxidation products like H_2_O, CO_2_, N_2_, and NO was detected, and SO_2_ appeared, which confirms the reduction of persulfate ions as well. After the complete decomposition of urea ligands (330 °C), the solid residue consisted of FeSO_4_ and Fe_2_(SO_4_)_3_. Thus, the urea or its decomposition products partially reduced iron(III) into iron(II).^17^

## 6 Formation of Simple and Mixed Metal-Oxide-Containing Phases during the Thermal Decomposition of Hexakis(urea)iron(III) Complexes

The heat treatment of the hexakis(urea)iron(III) complexes having oxidizing anions initiates a heat-induced quasi-intramolecular solid-phase redox reaction resulting in nanometer- or micrometer-sized simple or mixed oxides.^1,17,31,70,71^ Carp et al. and Zhao et al. found that the thermal decomposition of hexakis(urea)iron(III) nitrate at 800 °C in inert or oxidative atmospheres resulted in the formation of α-Fe_2_O_3_, however, in an argon atmosphere, Fe_3_O_4_ was found as an intermediate at 200 °C (see Figure 6a).^31,69,70^ The maghemite phase between 200 and 300 °C contained Fe^II^ and Fe^II^ ions and had an inverse spinel structure determined by IR and UV–vis measurements by Carp et al.^31^ The amount of this phase was 13.7%. The presence of Fe^II^ in the nanosized poorly crystallized green rust phase was attributed to the reduction of the iron(III)-containing components with urea or urea decomposition products due to insufficient oxygen for the complete oxidation of urea.^31^ A superparamagnetic intermediate with 34.0% iron(III) content (Figure 6a) was formed. Still, its Fe^III^ content decreased to 18% with the increase of the heating rate from 1.5 to 3.0 °C min^-1^ and if the calcination time was increased. The maghemite phase disappeared and transformed into hematite Figure 6a. During the decomposition of the peroxydisulfate complex (compound **7**), the final product of the heat treatment was α-Fe_2_O_3_ in both inert and oxidative atmospheres as well.^17^ The following eq 6 can describe the decomposition:In an oxidative atmosphere:
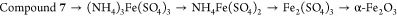
6In the inert atmosphere, the partial reduction of Fe^III^ centers occurred, and (NH_4_)_2_Fe(SO_4_)_2_ and NH_4_Fe(SO_4_)_3_ are formed. The evolution of H_2_O and CO_2_ as oxidation products was found at 200 °C.^17^ The further decomposition steps belong to the known decomposition routes of the ammonium iron(II) and iron(III) sulfates.^72−75^ The following eq 7 can describe the decomposition:In an inert atmosphere:
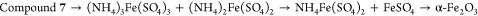
7The hexakis(urea)iron(III) permanganate (compound **5**) is an exciting member of this compound family because the anion is metal-containing and porous, and nanosized mixed iron–manganese oxide was formed in its thermal decomposition (Figure 6b).^1^ Béres et al. conducted a detailed study on the decomposition of compound **5** in both inert and oxidative atmospheres (Figures 6b and 7). Significant differences were found between the decomposition routes of compound **5** in the inert and the oxidative atmospheres. First of all, the heat treatment intermediates at 350 °C are single-phase (bixbyite, (Fe^III^,Mn^III^)_2_O_3_) and multiphase ((Fe^III^,Mn^III^)_2_O_3_ and (Mn^II^,M^T-4^(M,Mn^II^)^OC-6^)_2_O_4_ (M = Fe^III^,Mn^III^ in the spinel structure the T-4 (tetrahedra) site and the OC-6 (octahedral) site)) in the inert and oxidative (air) atmospheres, respectively.^1^ Additionally, a further redox reaction takes place in argon atmosphere between 300 and 600 °C, since NO (detected by TG-MS) was formed during heating, resulting in a wüstite-like compound ((Fe^II^,Mn^II^)O) (Figures 6(b) and 7).^1^ The Fe^II^ and Mn^II^ content was produced via the redox reaction between the amorphous iron–manganese mixed oxides intermediate and the organic decomposition residue of the urea ligand in the inert atmosphere.^1^ The final products of the heat treatment were the same as those in an inert atmosphere, and the experiment was conducted in air until 350 °C and then in N_2_ until 800 °C (Figures 6b and 7).^1^ This means the reduction of Fe^III^ into Fe^II^ took place above 350 °C. As in the thermal decomposition of compound **1**,^31^ the composition of the residue formed in the oxidative atmosphere changed with increasing calcination temperature and time. Two different bixbyite compositions were confirmed by Mössbauer spectroscopy and HR-TEM.^1^

## 7 Application of Hexakis(urea)iron(III) Complexes

### 7.1 As Precursor Materials in the Preparation of Simple or Mixed Transition Metal Oxides

#### 7.1.1 γ-Fe_2_O_3_

**Figure 8:**
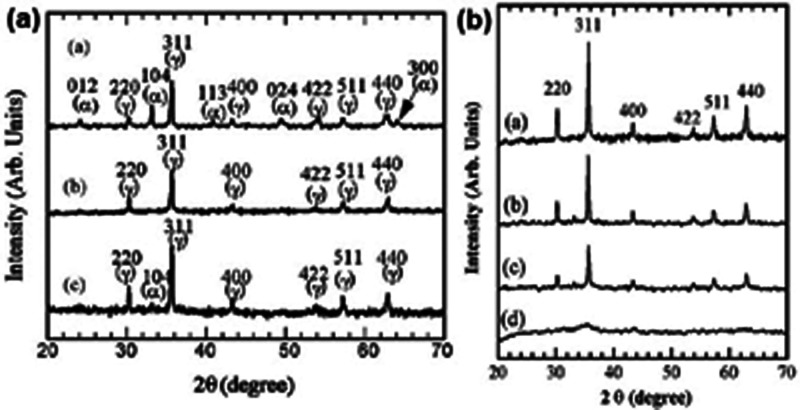
PXRD of the final products of direct thermal preparation of γ-Fe_2_O_3_ (a) without and (b) with CTAB. (a) Reprinted with permission from ref (78). Copyright 2009 Elsevier B.V. (b) Reproduced with permission from ref (71). Copyright 2012 Elsevier Masson SAS.

Zhao et al. and Asuha et al. applied direct thermal decomposition of the [Fe(urea)_6_](NO_3_)_3_ (compound **1**) to prepare γ-Fe_2_O_3_ by reproducing the results reported by Carp et al.:^31^ an isothermal heat treatment of compound **1** at 200 °C was done for a 1 h calcination time in air.^69,70,76,77^ The final product was phase-pure γ-Fe_2_O_3_ with an average of ∼24 nm grain size (XRD) (Figure 8a). The SEM and TEM analysis showed the formation of grains with spherical shape and size range of 20–50 nm.^69,70,76^ The γ-Fe_2_O_3_ prepared in this way was found to be ferromagnetic (212 Oe), and the permanent magnetization was 16.8 emu·g^–1^. Zhao et al. found that even a slight excess of free Fe^III^ ion initiates α-Fe_2_O_3_ formation during the calcination (Figure 8a).^69^ Asuha et al. found the same results in the experiments with compound **1** synthesized in the solid phase reaction of iron(III) nitrate nonahydrate and urea.^77^ Asuha et al. studied the effect of cetrimonium bromide (CTAB, cetyltrimethylammonium bromide) as a structure-directing agent on the decomposition of compound **1** according to Zhao’s experiments (200 °C, 1 h).^78^ The increased CTAB content decreased the crystallite size and increased the amorphicity (Figure 8b) and the BET surface area of the formed γ-Fe_2_O_3_.^78^ With the use of 15% CTAB, a completely amorphous, nanosized (<5 nm), and mesoporous (<3.4 nm pore size) γ-Fe_2_O_3_ was formed, which had superparamagnetic behavior and 149.8 m^2^·g^–1^ BET surface area.^78^ This mesoporous γ-Fe_2_O_3_ was a good absorber (about 95% perfectness under 30 min) of F^–^ ions at pH ∼ 3 from aqueous solutions. The maximum absorption capacity was found to be 7.9 m^2^·g^–1^. After three cycles, the absorption capacity was decreased to around 83%.^78^

**Figure 9:**
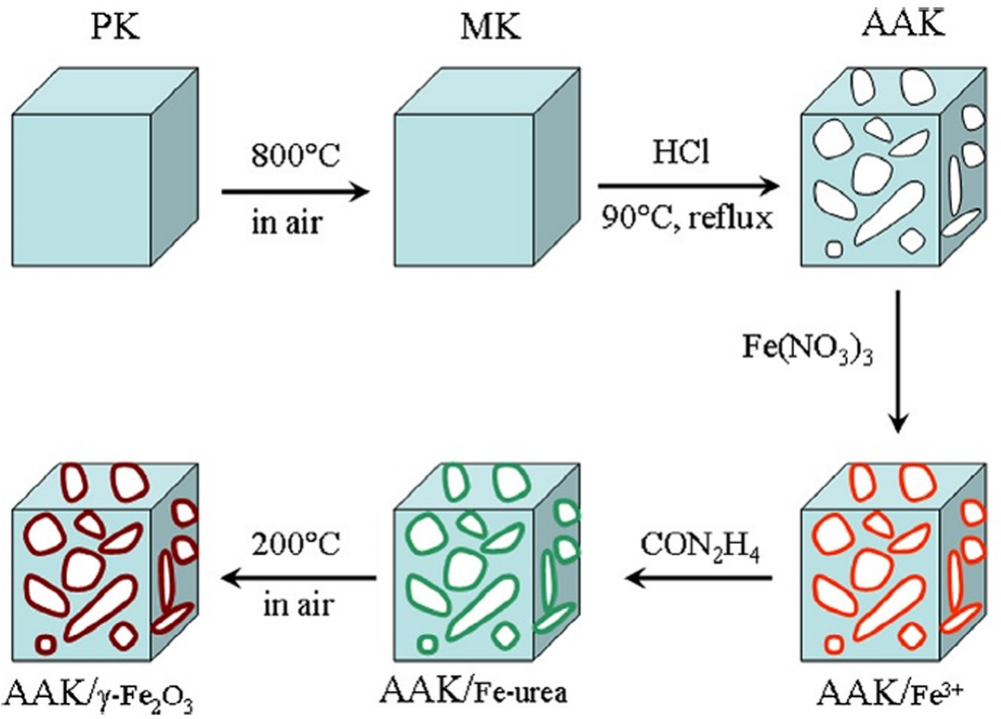
Synthesis of γ-Fe_2_O_3_ on the surface of acid-activated kaolin. Reprinted with permission from ref (79). Copyright 2014 Elsevier.

Gao et al. and Chang et al. found an easy way to produce nanometer-size (∼18 nm) γ-Fe_2_O_3_ particle-covered acid-activated kaolin (AAK/γ-Fe_2_O_3_) via the thermal decomposition of compound **1** by the method from Zhao et al. (200 °C, one h).^79,80^ The whole procedure is summarized in Figure 9. The formation of the AAK/compound **1** precursor (after step 4, Figure 9) was confirmed by IR spectroscopy. The BET surface of the AAK/γ-Fe_2_O_3_ was found to be 99.4 m^2^·g^–1^, and the material showed ferromagnetic characteristics.^79^ The absorbing ability of the material was tested with methylene blue (MB). The absorption maxima were almost reached after 5 min and found around 98% in pH-neutral circumstances (under pH = 5, it drops to 76%).^79^

Sharma et al. used a solvothermal route to prepare micrometer-size (1.2 ± 0.3 μm) γ-Fe_2_O_3_ from compound **1** under 35 min at 200 °C when a mixture of diphenyl ether and dimethylformamide was used as solvents.^80^ Further direct heat treatment of the solvothermal decomposition residue at 500 °C resulted in the formation of α-Fe_2_O_3_ with the same particle size.^80^ Sharma et al. used this reaction process to prepare ferromagnetic iron oxide@Ag core–shell nanoparticles with 52.8 ± 6.8 nm size by adding silver before the final direct heat treatment at 500 °C.^81^ The iron oxide@Ag core–shell nanoparticles were catalytically active in a reduction reaction of 4-nitrophenol and methylene blue aqueous solution with sodium borohydride. After 6 min, almost all 4-nitrophenol and all the methylene blue were reduced.^82^ Mahajan et al. used a modified method of Sharma et al.^71^ and Yu et al. used a direct thermal method to prepare TiO_2_@α-Fe_2_O_3_ core–shell heteronanostructures^83^ and γ-Fe_2_O_3_–TiO_2_^84^ from compound **1**, respectively. Mahajan et al. added TiO_2_ microspheres after the solvothermal treatment, and the calcination at 500 °C resulted in TiO_2_@α-Fe_2_O_3_ with about 368 ± 27 nm size.^83^ The photocatalytic activity of TiO_2_@α-Fe_2_O_3_ in the photodegradation of rhodamine B (in aqueous solution) with sunlight was tested. The degradation was between 85.8 and 98.0%.^83^

Yao et al.^85^ and Gao et al.^86^ used hexakis(urea)iron(III) chloride (compound **2**), while Bies et al.^4,87^ used the nitrate salt (compound **1**) to prepare different γ-Fe_2_O_3_-containing pigments with direct thermal^85,86^ and solvothermal^4,87^ heat treatment reaction routes.

#### 7.1.2 Fe_3_O_4_

Asuha et al. found an easy way to prepare Fe_3_O_4_ nanopowder in the direct thermal decomposition of compound **1**(88) in a closed vessel, the conditions of which favored the formation of Fe_3_O_4_ instead of γ-Fe_2_O_3_.^88^ The size of the particles depended on the calcination temperature: 37, 42, and 50 nm grains formed at 200, 250, and 300 °C at the same 2 h calcination time, respectively.^88^ The formed Fe_3_O_4_ powders proved ferromagnetic with large saturation magnetization (70.7, 79.4, and 89.1 emu·g^–1^ for 200, 350, and 300 °C calcination temperatures, respectively).^88^ The possible application fields of Fe_3_O_4_ prepared in this way were tested in biotechnology as biomedicine, although the size was too large to make dispersions in H_2_O or ethanol.^88^

Zhao et al. described a convenient preparation route of the nanosized Fe_3_O_4_ powders from compound **1** via solvothermal decomposition in ethanol.^69,89^ The heat treatment was done at 200 °C for 10, 30, and 50 h. As the calcination time increased, the material’s crystallinity decreased, whereas the average size increased to 9.7, 13.8, and 20.5 nm, respectively.^69^ The saturation magnetization values of the Fe_3_O_4_ formed increased with the particle size.^69^ With a similar solvothermal (but in ethylene glycol) method, Guan et al. prepared magnetic, nanosized (200 nm), and spherical Fe_3_O_4_ particles from [Fe(urea)_6_]Cl_3_ at 198 °C under 24 h. The BET surface area (N_2_) was 16.251 m^2^·g^–1^.^90^ The concentration of compound **2** in the solvent influenced the particle shape of the final product, as below 53.07 mmol·L^–1^ concentratio rather plate-like Fe_3_O_4_ particles formed.^90^ To increase the decomposition temperature to 260 °C, Asuha et al., Wan et al., and Wurendaodi et al. used triethylene glycol (TEG) as a solvothermal medium when a phas- pure superparamagnetic, nanosized Fe_3_O_4_ was formed.^3,91,92^ Asuha et al. found that after a 5 h reaction time the pore size and BET surface area were ∼3.6 nm (mesomorphous) and 122.0 m^2^·g^–1^, respectively.^3^ Surprisingly, the Fe_3_O_4_ prepared in TEG was dispersible in water and ethanol and kept its colloid behavior for several months due to the leftover hydrophobic TEG film on the surface.^3^ Wan et al. followed the influence of the calcination time on the structure of Fe_3_O_4_. After 20 h, all diffraction peaks of Fe_3_O_4_ appeared, and the magnetic saturation increased substantially from 21.4 to 48.5 emu·g^–1^.^91^ Keeping the 5 h calcination time and changing the concentration of compound **1** in TEG (8, 16, and 24 w%), the magnetic saturation was found to be 44.4, 50.6, and 53.8 emu·g^–1^, respectively. In contrast, the BET-specific surface area (N_2_) was 140 m^2^·g^–1^ in all cases.^91^ Wan et al. did a Cr^VI^ absorption test with the sample made at 16% compound **1** in TEG and a 5 h calcination time: the equilibrium was reached after 30 min, the absorption maxima was found to be 83%, and the maximum absorption capacity was 21.6 mg·g^–1^.^91^ Wurendaodi et al. used a modified method of Asuha et al. three developed for the preparation of a water-dispersible γ-Fe_2_O_3_:^92^ after the 5 h calcination at 260 °C, they did not use oxygen bubbling to oxidize the formed Fe_3_O_4_ into γ-Fe_2_O_3_. The BET surface area was found to be 102.0 m^2^·g^–1^.^92^

Cappelletti et al. prepared Pd-containing core–shell superparamagnetic (SPNS) Fe_3_O_4_ nanoparticle catalysts (Fe_3_O_4_@Pd-OA) via solvothermal decomposition of compound **1** in a mixture of oleylamine and dibenzyl ether at 300 °C by seeding SPNPs-Fe_3_O_4_ heat-treated with Pd(Acac)_2_ at 200 °C in the same mixture.^93^ The average size of the 1.25–1.35 nm thick Pd shell-covered SPNS-Fe_3_O_4_ was 5.1 ± 0.1 nm, and it had good catalytic activity in the Suzuki–Miyaura coupling reaction of *p*-iodoanisole with boronic acids (>93% conversion and >80% isolated yield).^93^

#### 7.1.3 Mixed Oxides

Hexakis(urea)iron(III) chloride (compound **2**) was used as an iron source in the preparation of nanometer-sized zinc ferrite from the mixture of compound **2**, Zn(NO_3_)_2_·6H_2_O or Zn(CH_3_COO)_2_·2H_2_O, and α- or β-cyclodextrin with heat treatment of the obtained light yellow solid intermediate at 600–650 °C for 2–6 h. The nanozinc ferrite yields were 75.6% and 82.3% with the usage of Zn(NO_3_)_2_·6H_2_O/ α-cyclodextrin and Zn(CH_3_COO)_2_·2H_2_O/β-cyclodextrin Zn precursors, respectively.^94^

**Figure 10:**
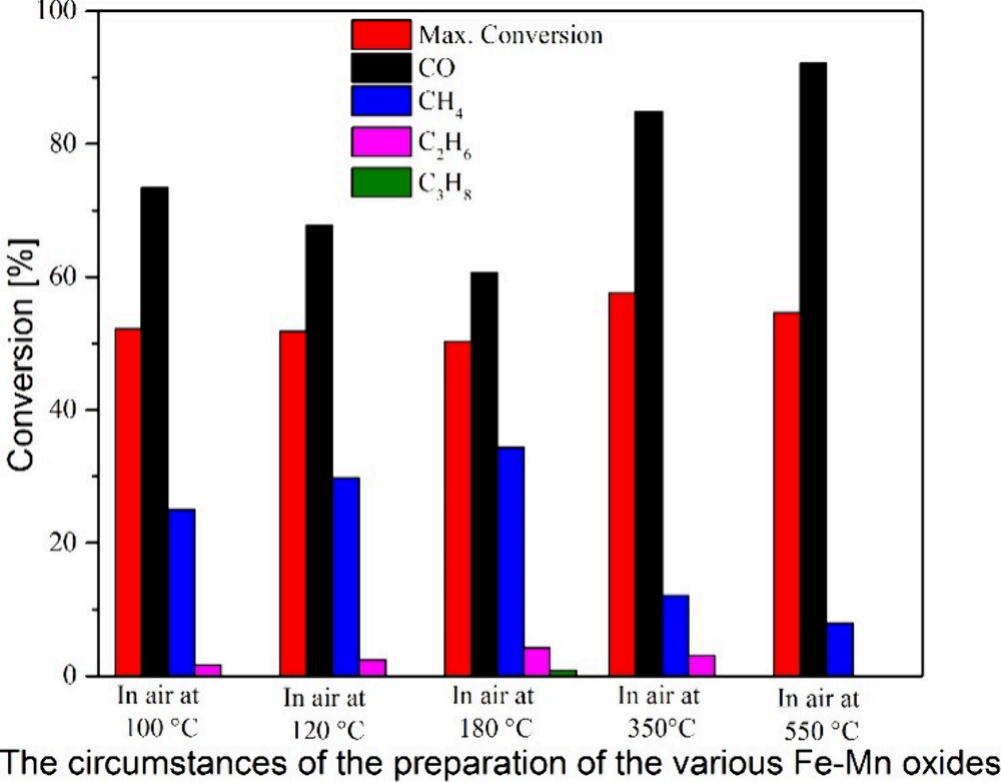
Catalytic effect of the thermal decomposition products of [Fe(urea)_6_](MnO_4_)_3_ in CO_2_ hydrogenation. Reprinted with permission from ref (1). Copyright 2022 American Chemical Society.

A series of iron manganese mixed oxides prepared from the permanganate compound (compound **5**) were prepared under various experimental conditions (calcination atmospheres, temperatures, and times).^1^ The amorphous Fe–Mn oxides formed between 120–350 and 120–800 °C (in oxidative and inert atmospheres, respectively), and the phase pure bixbyite-like Fe–Mn oxides prepared in air at 800 °C for various calcination times, as well as wüstite-like Fe–Mn oxides prepared in an inert atmosphere at 800 °C for 2 h, were all tested in the CO_2_ hydrogenation reaction. (Figure 6b and 7).^1^ Their catalytic activity was studied in CO_2_-rich conditions (H_2_ to CO_2_ ratio was 3:1) at 20 bar between 175 and 550 °C for four h. The overall conversion of CO_2_ over 350 °C was between 50–60% (Figure 10). The main reaction products were CO, CH_4_, and C_2_H_6_, but even C_3_H_8_ was formed at 180 °C.^1^

### 7.2 The Preparation of Fe_2_N and Carbon Nanotubes (CNTs) from Hexakis(urea)iron(III) nitrate

Jiang et al. prepared an electrically conductive improved carbon nanotube (CNT)–magnetite nanocomposite with solvothermal heat treatment of a hexakis(urea)iron(III) nitrate and CNT mixture in ethylene diamine at 200 °C for 50 h.^2^ The final product of the heat treatment depended on the starting compound **1** to CNT ratio, temperature, and time. Magnetite–hematite–CNTs or hematite–CNTs were formed. The results are summarized in Table 4.^2^

**Table 4:** Final Products of the Heat Treatments of Hexakis(urea)iron(III) Nitrate and CNT Mixture^a^

weight ratio Fe[(NH_2_)_2_CO]_6_(NO_3_)_3_/CNTs	solvent	temp (°C)	time (h)	product containing CNTs
10:1	C_2_H_8_N_2_	100	50	unreacted precursor
10:1	C_2_H_8_N_2_	150	50	α-Fe_2_O_3_ + unreacted precursor (trace)
10:1	C_2_H_8_N_2_	200	10	α-Fe_2_O_3_ + Fe_3_O_4_
10:1	C_2_H_8_N_2_	200	25	α-Fe_2_O_3_(trace) + Fe_3_O_4_
10:1	C_2_H_8_N_2_	200	50	Fe_3_O_4_
without CNTs	C_2_H_8_N_2_	200	50	α-Fe_2_O_3_(trace) + Fe_3_O_4_ (without CNTs)
20:1	C_2_H_8_N_2_	200	50	Fe_3_O_4_
5:1	C_2_H_8_N_2_	200	50	α-Fe_2_O_3_(trace) + Fe_3_O_4_
2:1	C_2_H_8_N_2_	200	50	α-Fe_2_O_3_(trace) + Fe_3_O_4_
1:1	C_2_H_8_N_2_	200	50	α-Fe_2_O_3_ + Fe_3_O_4_
10:1	C_2_H_5_OH	200	50	α-Fe_2_O_3_
10:1	H_2_O	200	50	α-Fe_2_O_3_
10:1 (baked CNTs)	C_2_H_8_N_2_	200	10	α-Fe_2_O_3_ + Fe_3_O_4_
10:1 (baked CNTs)	C_2_H_8_N_2_	200	25	α-Fe_2_O_3_ + Fe_3_O_4_
10:1 (baked CNTs)	C_2_H_8_N_2_	200	50	α-Fe_2_O_3_(trace) + Fe_3_O_4_

a Reprinted with permission from ref (2). Copyright 2003 American Chemical Society.

**Figure 11:**
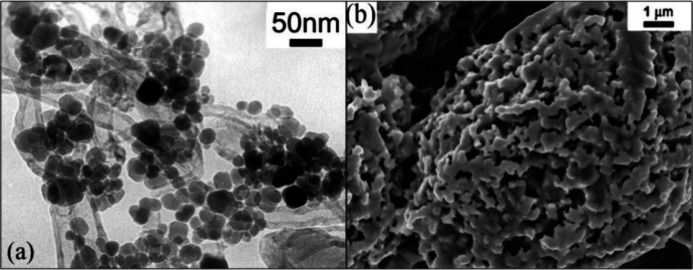
(a) TEM micrograph of the pure magnetite–CNT nanocomposite and (b) SEM of Fe_2_N synthesized at 600 °C for one h. (a) Reprinted with permission from ref (2). Copyright 2003 American Chemical Society. (b) Reprinted with permission from ref (95). Copyright 2004 The American Ceramic Society.

The 10:1 ratio of compound **1** to CNTs resulted in pure magnetite–CNT nanocomposite with 20–30 nm particle size in 50 h (Figure 11a).^2^ The specific surface area of the product was found to be 58.7 m^2^·g^–1^, whereas its electrical conductivity (σ) was 2.5 S·cm^–1^ (for simple CNT, it was 1.9 S·cm^–1^).^2^

Qiu et al. prepared ζ-Fe_2_N with the direct thermal decomposition of compound **1** under an NH_3_ atmosphere at various calcination temperatures and times. Still, the lowest temperature was fruitful at 600 °C with a 1 h calcination time.^95^ The resulting Fe_2_N was formed as a porous, sponge-like structure containing large holes and voids (Figure 11b).^95^

### 7.3 Hexakis(urea)iron Complex Reagents and Catalysts in Organic Reaction

The properties of the complex cation with six urea ligands have an enormous influence on the properties of the counterions, e.g., their oxidation ability or reactivity toward other materials. Since the urea ligands give organic-like behavior to the complex salts, they can easily be used in different organic media as reagents or catalysts. For example, the chloride compound (compound **2**) was used with high efficiency as a catalyst in the reduction of aldehydes into alcohols with sodium borohydride (Table 5).^96^

**Table 5:**
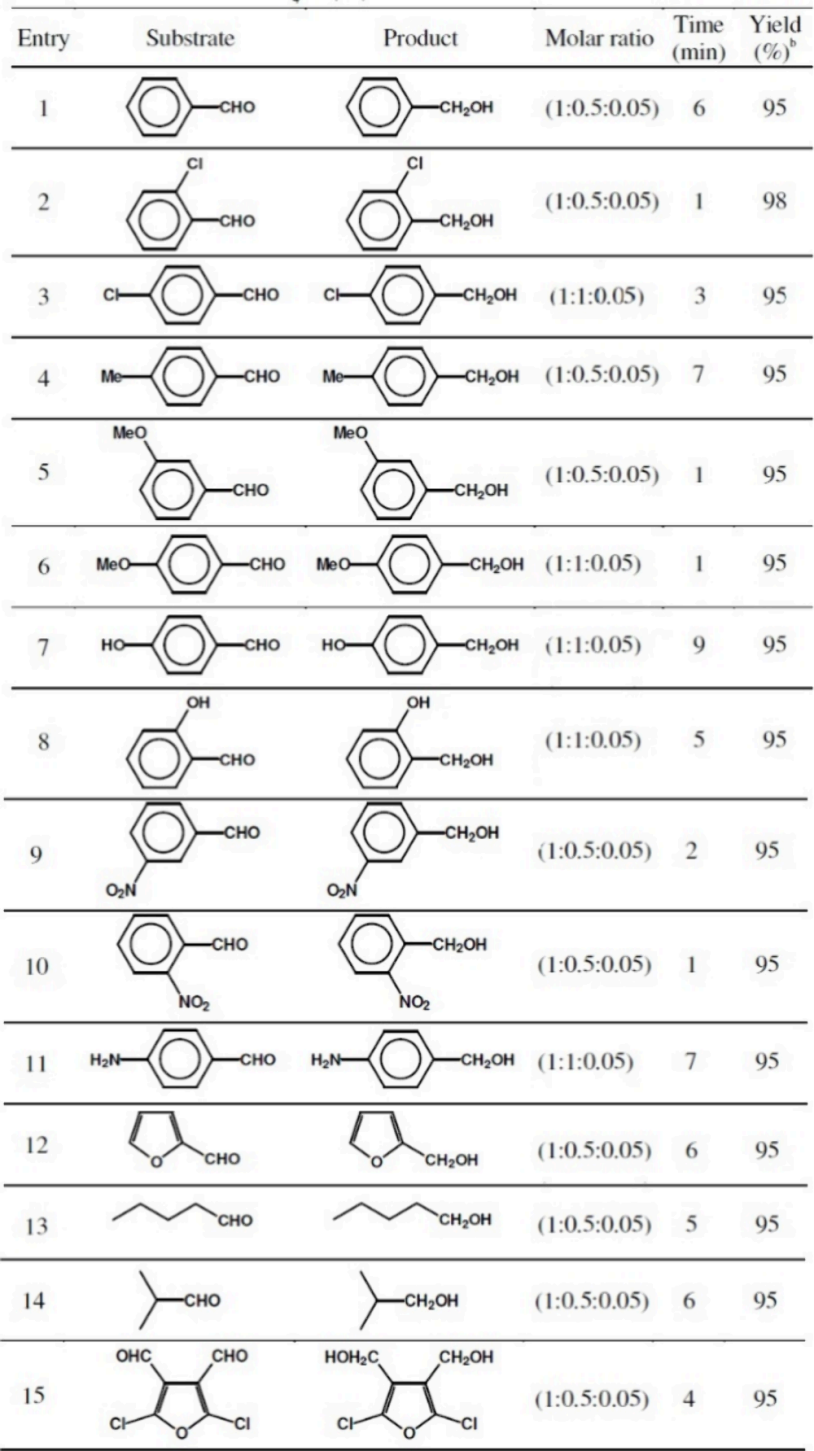
Reduction of Aldehydes to Alcohol with NaBH_4_/Compound **2** System^c^

a All reactions were performed in CH_3_CN at room temperature.

b Yields refer to isolated pure products.

c Reprinted with permission from ref (96). Copyright 2008 ASIAN PUBLICATION CORPORATION.

The reduction of carbonyl compounds (aldehydes, ketones, conjugated carbonyl compounds, α-diketones, and acyloins) into the corresponding alcohols was performed. The reaction of aldehydes was very fast (1–10 min) in CH_3_CN at room or reflux temperature (82 °C) and resulted in ∼95% yields.^96^

The secondary alcohols were formed from ketones in at least 96% yield (Table 6), except the 1-phenylethanol from acetophenone, which was formed only in 75% yield even after 1.5 h of reflux.^96^ The vicinal diols were formed from α-diketones and acyloins in 96–98% yields (Table 7).^96^

**Table 6:**
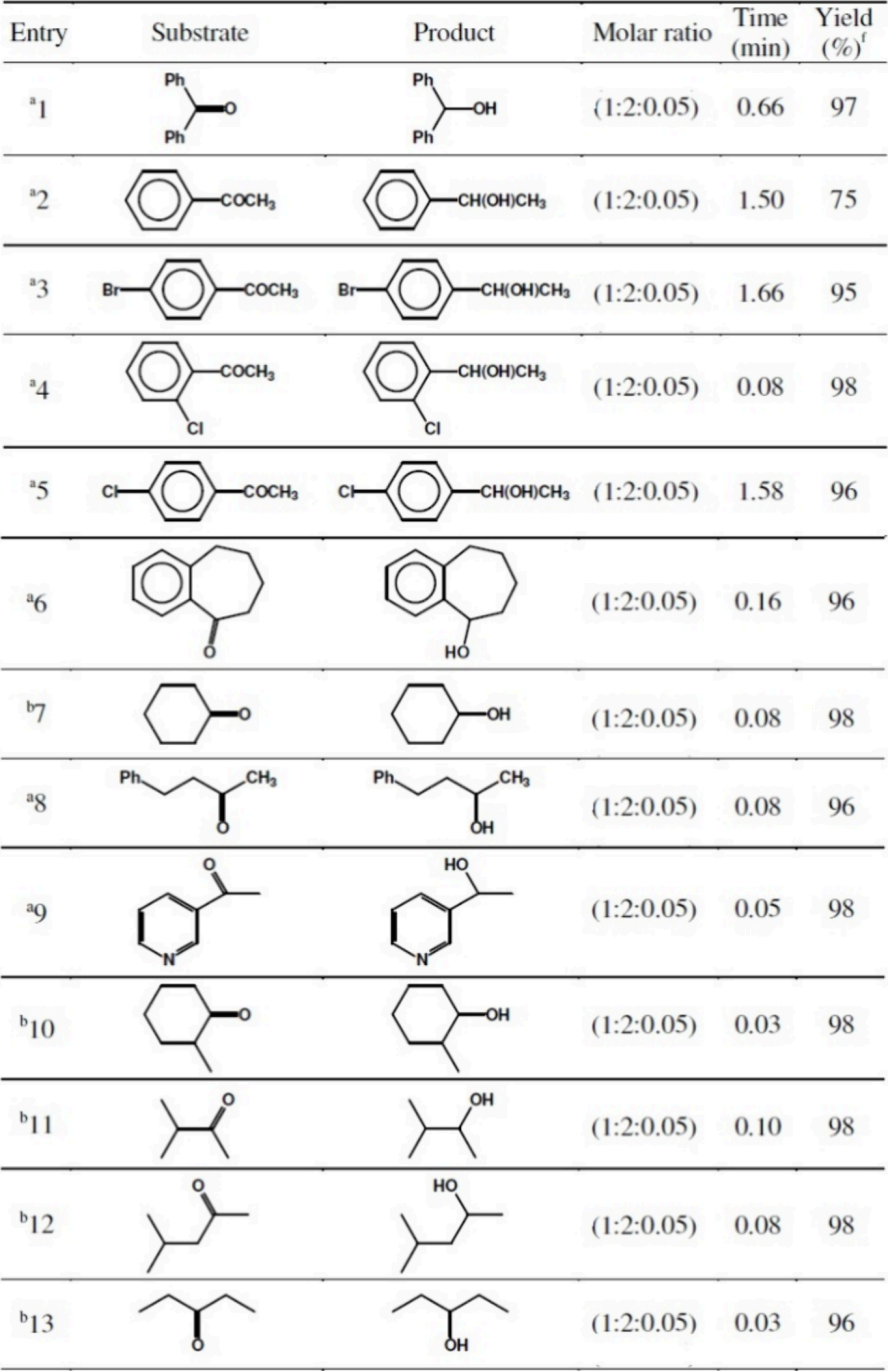
Reduction of Ketones to Alcohol with NaBH_4_/Compound **2** System^f^

a All reactions were performed in CH_3_CN under reflux conditions.

b All reactions were performed in CH_3_CN at room temperature.

c Yields refer to isolated pure products.

f Reprinted with permission from ref (96). Copyright 2008 ASIAN PUBLICATION CORPORATION.

**Table 7:**
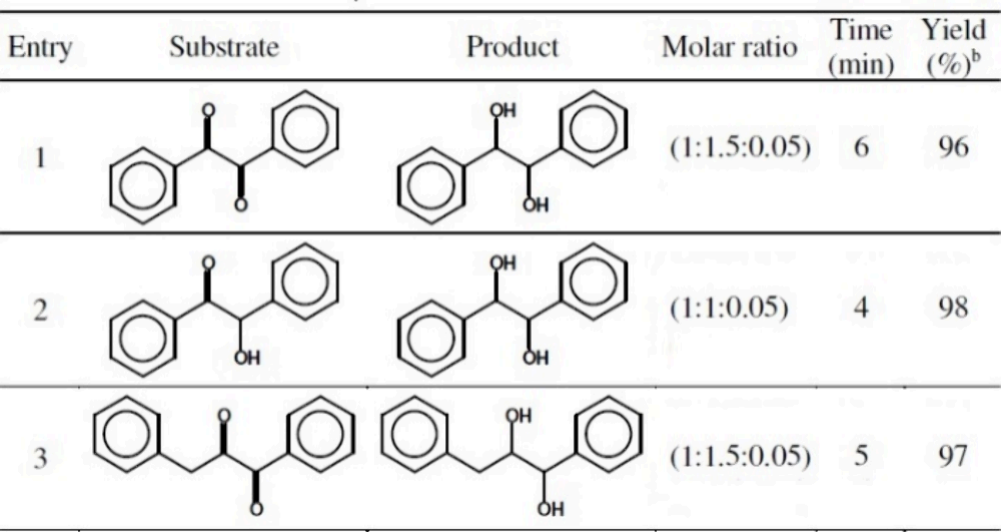
Reduction of Ketones to Alcohol with NaBH_4_/Compound **2** System^c^

a All reactions were performed in CH_3_CN at room temperature.

b Yields refer to isolated pure products.

c Reprinted with permission from ref (96). Copyright 2008 ASIAN PUBLICATION CORPORATION.

The compound **2**–NaBH_4_ mixture showed very promising 1,2-regioselectivity in the reduction of α,β-unsaturated aldehydes and ketones, and the products were allylic alcohols with high yields, even at room temperature (95–98%) (Table 8).^96^ However, the reduction of unsaturated ketones required more harsh conditions to reach excellent yields (94–98%) (Table 8).^96^

**Table 8:**
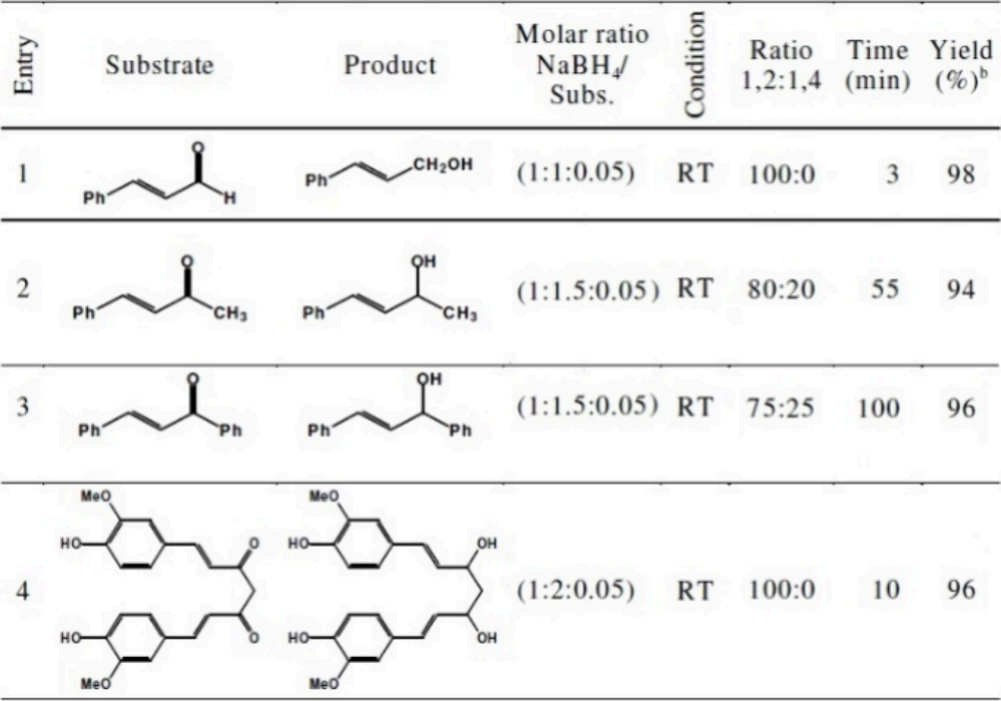
Reduction of Conjugated Carbonyl Compounds with NaBH_4_/Compound **2** System^c^

a All reactions were performed in CH_3_CN at room temperature.

b Yields refer to isolated pure products.

c Reprinted with permission from ref (96). Copyright 2008 ASIAN PUBLICATION CORPORATION.

Zhang et al. used the hexakis(urea)iron(III) nitrate, chloride, and sulfate as cocatalysts in the oxidation of cyclohexane over *N*-hydroxyphthalate Imide (NHPI) in acetonitrile under 0.6 MPa O_2_ at 100 °C for 6 h (Table 9).^97^ The main reaction products were cyclohexanol, cyclohexanone (KA oil), adipic acid, and a small amount of glutaric acid.

**Table 9:** Summarized (Co)Catalytic Results of Compounds **1**, **2**, and **9** in the Oxidation Reaction of Cyclohexane over NHPI

catalyst	conversion rate (%)	KA oil yield (%)	acid yield (%)
compound **2**	1	1	
compound **1** + NHPI	37	14	23
compound **2** + NHPI	36	20	16
compound **9** + NHPI	39	16	23

There was no conversion of cyclohexane observed without NHPI. Still, the cocatalyst systems resulted in ∼40% conversion rates and varied selectivity depending on the anion in the hexakis(urea)iron(III) complexes (Table 9).^97^ The low conversion rate was attributed to the low amount of the high-valent iron-containing active intermediates with a higher oxidation state than 3–.

It is well-known that the complex cations modify the oxidation power of the complexed transition metal permanganates.^1,61,98,99^ Accordingly, the urea ligand-complexed iron(III) permanganate^1^ was the first example of iron permanganates, which could be used in organic synthesis. Hexakis(urea)iron(III) permanganate (compound **5**) as a heterogeneous oxidant in benzene selectively oxidized different benzyl alcohols (R–C_6_H_4_CH_2_OH) at room and reflux temperatures^1^ into benzaldehydes and benzonitriles without the formation of benzoic acids (Table 10).^1^

**Table 10:** Oxidation of Benzyl Alcohols (R–C_6_H_4_CH_2_OH) into Benzaldehydes and Benzonitriles with Compound **5** in Benzene^a^

			conversion of benzyl alcohols		
R-C_6_H_**4**_CH_2_OH	time (h)	temperature	R–C_6_H_4_C(O)H	R–C_6_H_4_CN	unconverted
R = H	2	25 °C	76		24
	2	reflux	93	7	
	4	reflux	68	32	
	4	reflux	60	36	2
	11	reflux	21	77	
R = **2**-I	2	25 °C	29		71
	2	reflux	79	3	18
	4	reflux	64	14	22
R = **2**-NO_2_	2	25 °C	13		87
	4	reflux	37	63	
R = **2**-MeO	2	25 °C	60		40
	2	reflux	62	11	24
	4	reflux	57	32	11
R = **4**-NO_2_	2	25 °C	100	0	
	4	reflux		100	

a Reprinted with permission from ref (1). Copyright 2022 American Chemical Society.

The presence and position of the substituents also had an enormous influence on the aldehyde/nitrile formation and conversion. For example, a strong electron-withdrawing NO_2_ group, depending on the reaction conditions, resulted in either aldehyde (at room temperature) or nitrile (reflux temperature) with quantitative yields (Table 10).^1^ A similar nitrile formation reaction was observed only in the oxidation of benzyl alcohol with NH_4_MnO_4_.^60^ It is strongly suggested that ammonia formation took place from the urea ligand of compound **5**, which cannot bind strongly to the iron central atom because the strongly bound ammonia in the reaction of tetraamminecopper(II) permanganate resulted in only a tiny amount of benzonitrile.^100,101^

### 7.4 Utilization of Hexakis(urea)iron(II) Iodide as an Elementary Iodine Absorber

Zhiveinova et al. studied the utilization of hexakis(urea)iron(II) iodide (compound **10**) as an elementary iodine impurity absorber from a gaseous mixture^20^ according to the polyiodide salt formation found by Savinkina et al. when the iodine was reacted with the solution of compound **10** with a polyiodide complex (compound **11**).^19^ Under static conditions, 60.0 wt % of the iodine was absorbed.^20^ A similar result was found in dynamic conditions when compound **10** was mixed with glass wool in different ratios. The best absorption rate was found when the compound **10** to carrier ratio was between 67:33 and 75:25 wt %. In both cases, at 3.0, 65.0, and 227.7 mg/L iodine content of the air, the absorption of iodine was around 60%.^20^

### 7.5 Utilization of Hexakis(urea)iron Complexes in Agriculture: Raw Material for Fertilizers

Iron is known to be an essential trace element for plants since it is involved in crucial procedures (like various cellular functions) for plants’ development and growth. Therefore, agricultural and fertilizer intake studies involving iron are the focus of many investigations.^102,103^ Hexakis(urea)iron(III) nitrate and chloride (compounds **1** and **2**) are widely used as stable and slow releasing of nitrogen and iron sources in fertilizers.^5,24,104,105^ Chein et al. found that if the urea is fixed in different metal–urea complex salts, like hexakis(urea)iron(III) nitrate, the NH_3_ volatilization losses from soils can be reduced compared to the normal urea usage.^105^ Furthermore, if the urea complex salt of iron(III) nitrate was used, the free water content could be kept between 1.28% ad 2.08%, which helps prevent the deterioration of the fertilizer products during 6 weeks of storage.^105^ A mixture of compound **1** and monocalcium phosphate monohydrate (MCP*H_2_O) or triple superphosphate (TSP) was also used as a phosphate carrier.^105^ Chen et al.^24^ and Cao et al.^5^ described compound **1** as an excellent trace element fertilizer and long-lasting nitrogen fertilizer and proposed its use as a possible low-cost raw material.

Moreover, its production method is suitable for industrial circumstances. Zhu et al., Yuan et al., and Xin et al. used compound **1** as an additive in a fertilizer made from organic waste. The urea complex was used as chelating material containing trace elements (like iron).^107−109^ Wang et al. used compound **1** as an inert part in a foliar fertilizer, which has rambutan-shaped hollow mesoporous SiO_2_ balls. This material is capable of adsorbing water-soluble cationic plant nutrients like compound **1**.^106^

Compound **1** in rice cultivation resulted in high water and fertilizer-conserving capacity.^110^ Compound **1** was added as a high-utilization-rate fertilizer specialized for wheat as a trace element additive, which simultaneously increased the soil’s water-keeping ability, quality, and fertility as well.^111−115^ Compound **1** was used as nitrogen and trace element fertilizer for fruits like peaches,^116−118^ grapes, and strawberries^119−121^ and as a slow-releasing fertilizer for grapefruit.^122^ Similarly, compound **1** is a slow-release trace element root fertilizer additive for tomato,^123^ radish,^124^ eggplant,^125^ cauliflower,^126^ lettuce,^127^ and chili.^128−130^ In the case of tea and stevia rebaudiana, the well-grown and high-yield leaves are essential, and due to the long life cycle compound **1** as a slow-release fertilizer had good results.^131,132^

Compounds **1** and **2** were tested as fertilizers for ornamental trees and plants, like lotus or sakura trees. It is of enormous importance to use fertilizers for lotus trees to improve the roots’ quality by ensuring trace elements;^133^ however, due to the wet life environment, only slow- and controlled-release compounds such as the hexakis(urea)iron complexes can be used to avoid a potentially environmental pollution.^134−137^ To ensure high-nutrition soils for sakura trees,^137^ transplantation of holly trees^138^ or garden seedlings,^136,139^ these kinds of fertilizers were used with high efficiency. For flowers, like *Camellia oleifera*,^140−142^ different kinds of roses,^143−145^ or ornamental plants like *Asparagus plumosus*(146,147) or *Epipremnum aureum*,^148^ a comprehensive nutrition content and high utilization rate were reached with the use of these complexes.

## 8 Conclusions

The comprehensive review of hexakis(urea)iron(II/III) complexes shows that the members of these versatile material families can easily be prepared in high yield. The overview of the structural, spectroscopic (IR, Raman, UV–vis, Mössbauer, EPR, and X-ray), and thermal properties shows our existing knowledge about hexakis(urea)iron(II/III) salts and gives an outlook for further studies of these complexes. It is concluded that almost all hexakis(urea)iron(II/III) salts are excellent precursors of nanosized porous iron oxides. The exchange of the outer-sphere anions to metal-containing ones (like permanganate) gives outstanding potential to prepare precursors for synthesizing mixed iron-transition metal oxides. Due to the solid-phase quasi-intramolecular redox reactions between the oxidizing anions and reducing urea ligands, these mixed oxides can be synthesized at significantly lower temperatures, which ensures the low crystallinity of the oxide products. The properties of these oxides can be adjusted on a broad scale, which opens new routes to prepare useful absorbers and catalysts in various industrially important processes like photodegradation of dyes or reduction of CO_2_. Furthermore, some hexakis(urea)iron(II/III) complexes can be used as reagents in different organic reactions. The urea content and variation of the anions ensure the outstanding potential to prepare valuable agricultural materials as selective fertilizers.
